# Malaria outbreak in Laos driven by a selective sweep for *Plasmodium falciparum kelch13* R539T mutants: a genetic epidemiology analysis

**DOI:** 10.1016/S1473-3099(22)00697-1

**Published:** 2023-05

**Authors:** Varanya Wasakul, Areeya Disratthakit, Mayfong Mayxay, Keobouphaphone Chindavongsa, Viengphone Sengsavath, Nguyen Thuy-Nhien, Richard D Pearson, Sonexay Phalivong, Saiamphone Xayvanghang, Richard J Maude, Sónia Gonçalves, Nicholas P Day, Paul N Newton, Elizabeth A Ashley, Dominic P Kwiatkowski, Arjen M Dondorp, Olivo Miotto

**Affiliations:** a Mahidol-Oxford Tropical Medicine Research Unit, Faculty of Tropical Medicine, Mahidol University, Bangkok, Thailand; b Lao-Oxford-Mahosot Hospital-Wellcome Trust Research Unit, Microbiology Laboratory, Mahosot Hospital, Vientiane, Laos; c Institute of Research and Education Development, University of Health Sciences, Ministry of Health, Vientiane, Laos; d Centre for Tropical Medicine and Global Health, Nuffield Department of Medicine, Oxford University, Oxford, UK; e Centre for Malariology, Parasitology, and Entomology, Vientiane, Laos; f Oxford University Clinical Research Unit, Ho Chi Minh City, Viet Nam; g Wellcome Sanger Institute, Hinxton, UK; h Harvard TH Chan School of Public Health, Harvard University, Boston, MA, USA

## Abstract

**Background:**

Malaria outbreaks are important public health concerns that can cause resurgence in endemic regions approaching elimination. We investigated a *Plasmodium falciparum* outbreak in Attapeu Province, Laos, during the 2020–21 malaria season, using genomic epidemiology methods to elucidate parasite population dynamics and identify its causes.

**Methods:**

In this genetic analysis, 2164 *P falciparum* dried blood spot samples were collected from southern Laos between Jan 1, 2017, and April 1, 2021, which included 249 collected during the Attapeu outbreak between April 1, 2020, and April 1, 2021, by routine surveillance. Genetic barcodes obtained from these samples were used to investigate epidemiological changes underpinning the outbreak, estimate population diversity, and analyse population structure. Whole-genome sequencing data from additional historical samples were used to reconstruct the ancestry of outbreak strains using identity-by-descent analyses.

**Findings:**

The outbreak parasite populations were characterised by unprecedented loss of genetic diversity, primarily caused by rapid clonal expansion of a multidrug-resistant strain (LAA1) carrying the *kelch13* Arg539Thr (R539T) mutation. LAA1 replaced *kelch13* Cys580Tyr (C580Y) mutants resistant to dihydroartemisinin–piperaquine (KEL1/PLA1) as the dominant strain. LAA1 inherited 58·8% of its genome from a strain circulating in Cambodia in 2008. A secondary outbreak strain (LAA2) carried the *kelch13* C580Y allele, and a genome that is essentially identical to a Cambodian parasite from 2009. A third, low-frequency strain (LAA7) was a recombinant of KEL1/PLA1 with a *kelch13* R539T mutant.

**Interpretation:**

These results strongly suggest that the outbreak was driven by a selective sweep, possibly associated with multidrug-resistant phenotypes of the outbreak strains. Established resistant populations can circulate at low frequencies for years before suddenly overwhelming dominant strains when the conditions for selection become favourable—eg, when front-line therapies change. Genetic surveillance can support elimination by characterising key properties of outbreaks such as population diversity, drug resistance marker prevalence, and the origins of outbreak strains.

**Funding:**

Bill & Melinda Gates Foundation; The Global Fund to Fight AIDS, Tuberculosis and Malaria; Wellcome Trust.

**Translation:**

For the Lao translation of the abstract see Supplementary Materials section.

## Introduction

The parasite *Plasmodium falciparum* is the causal pathogen of the deadliest form of malaria, which causes hundreds of thousands of deaths every year, the majority of which are children in sub-Saharan Africa. Over the past two decades, substantial progress has been made towards reducing this burden, largely through public health interventions.^1^, ^2^ Mortality has been greatly reduced thanks to highly efficacious artemisinin-based combination therapies (ACTs), which combine artemisinin derivatives, which are fast-acting parasiticidals, with a longer-lasting partner drug that clears surviving parasites. In the past decade, however, strains with reduced sensitivity to artemisinin have emerged and spread in the Greater Mekong Subregion, where transmission is low to moderate, and mortality is low.^3^, ^4^, ^5^, ^6^, ^7^ Some of these strains are also resistant to partner drugs (mefloquine and piperaquine),^8^, ^9^, ^10^ reducing ACT efficacy and forcing public health authorities to change front-line therapies. To protect ACT efficacy globally, and particularly in Africa, intense efforts have been undertaken to contain the spread of resistant strains and to eliminate *P falciparum* from the Greater Mekong Subregion within the next decade. Although these efforts have led to a reduction of malaria cases across the region, the resulting increased drug pressure has caused the emergence of new resistant forms, which present new public health challenges. It has become essential for Greater Mekong Subregion public health authorities to monitor epidemiological changes, such as localised malaria outbreaks, and respond appropriately to combat the resurgence of *P falciparum*.

**Research in context**

**Evidence before this study**

This study builds on previous work on the spread of multidrug-resistant *Plasmodium falciparum* strains in the Greater Mekong Subregion. Here, we sought to identify the driving forces of a recent outbreak in Attapeu Province, Laos. We searched PubMed, without language restrictions from database inception to June 30, 2022, for literature pertaining to the underlying characteristics of malaria outbreaks. A search for outbreaks associated with human movement using the terms: “malaria”, “outbreaks”, “transmission”, (“migration” OR “imported cases”), and “diversity” yielded 47 publications, including reports on high-diversity outbreaks caused by imported cases and recombination, and on parasite population shaped by migration of vectors and humans that maintain gene flows. Another search for the effects of genetic selection using the terms “malaria”, “selective sweep”, and “drug resistance” yielded 37 publications, including reports of reduced genetic diversity caused by selective sweeps of multidrug resistant malaria parasites.

**Added value of this study**

We analysed the genetic characteristics of outbreak parasites, using samples collected in Attapeu by routine surveillance. The outbreak was driven by the clonal expansion of LAA1, an artemisinin-resistant parasite population. Differently from the previously dominant KEL1/PLA1 strain, LAA1 carries the *kelch13* R539T allele and no markers of piperaquine resistance. The expansion of LAA1 bears the hallmarks of a selective sweep, coinciding with regional changes in drug pressures. Despite the presence of two other minor strains, LAA1 parasites did not outcross, suggesting their selective advantage might be diminished through recombination. A large portion of the genomes of the three outbreak strains, including the regions containing *kelch13*, can be traced back to parasites circulating in western Cambodia around 2008–10.

**Implications of all the available evidence**

This study shows that malaria outbreaks, frequently ascribed to environmental or behavioural causes, can be driven by genetic selection. The clonal expansion causing the Attapeu outbreak coincided with the end of dihydroartemisinin–piperaquine use in neighbouring countries, which weakened the drug pressure sustaining the KEL1/PLA1 strain. The Attapeu outbreak populations are close descendants of artemisinin-resistant lineages found before 2010 in Cambodia, which persisted over long periods at very low frequency. These lineages have been very resilient over time, and can rapidly dominate when finding a fitness niche such as a favourable change in front-line therapy. Therefore, it remains crucial to monitor all *kelch13* mutants; we have shown that genetic surveillance is a powerful tool to track these variants and explain the cause and dynamics of malaria outbreaks.

Malaria outbreaks are generally thought to be caused by increased transmission, which can be driven by one or more factors. These factors include human behaviour, such as increased travel to higher-transmission forested areas; changing ecology or climate affecting the mosquito population; or importation of parasites into areas where elimination had been achieved.^11^ A less frequently considered factor is genetic selection, which might occur when a parasite strain expands in numbers after acquiring a genetic trait that increases its chances to survive or to transmit. Genetic traits might favour transmission in various ways—eg, by improving adaptation to local vector species, or by increasing gametocyte production.^12^ Human populations in low-transmission regions such as the Greater Mekong Subregion develop lower levels of immunity than populations who are frequently bitten by infected mosquitoes. Hence, a higher proportion of infections progress to a symptomatic state and are treated with antimalarials, reducing the parasites' chances to transmit when the drugs are efficacious. Conversely, reduced drug sensitivity could allow more time for gametocytes to be produced before the infection is cleared.^3^, ^12^, ^13^

Analyses of genetic patterns in outbreak populations can be informative about the factors driving the outbreak. When genetic selection is the driver of an outbreak, offspring produced by self-fertilisation inherit the selected mutations and are more likely to survive, resulting in rapid expansions of identical individuals, and a collapse of genome-wide variation.^14^ Thus, an outbreak driven by selection might be characterised by reduced population diversity, decreased heterozygosity, and large clonal clusters.^5^, ^15^ In contrast, outbreaks driven by ecological or human factors would tend to maintain or increase the outcrossing rate, maintaining typical or increased heterozygosity and allelic diversity in the population.^16^ Therefore, in-depth analyses of parasite population structure changes can discriminate between different outbreak drivers, helping public health authorities decide on the most effective response—eg, whether to target forest workers, or investigate treatment efficacy.

Laos, a country in the Greater Mekong Subregion, is aiming to eliminate transmission of *P falciparum* by 2023. Since 2004, artemether–lumefantrine has been the first-line treatment for *P falciparum*,^17^ which is only endemic in five southern provinces, and case numbers have been steadily decreasing. However, during the 2020–21 malaria season, the province of Attapeu had a sudden increase in *P falciparum* malaria case numbers (130% increase; 378 cases in 2019 and 868 cases in 2020), at a time when other endemic provinces had a marked reduction (mean reduction of 65%). Attapeu is a largely forested province with low population density that borders with Cambodia and Viet Nam, with little international traffic. Malaria is mostly transmitted in the forest areas where *Anopheles* vectors circulate, and primarily affects forest workers and their communities. The GenRe-Mekong project has been conducting routine genetic surveillance of malaria in southern Laos since 2017 in collaboration with the Centre of Malariology, Parasitology, and Entomology of Laos, and collected 249 samples from Attapeu Province during the outbreak. These samples were genotyped with a broad set of markers,^18^ and their genetic signatures were compared with those of parasites from previous seasons and other geographical locations. In this study, by use of genetic analyses we aim to investigate the outbreak parasite population dynamics, characterise the Attapeu outbreak strains, and reconstruct their origins.

## Methods

### Sample collection

In this genetic analysis, between Jan 1, 2017, and April 1, 2021, 2078 *P falciparum* dried blood spot samples were collected at 55 local public health sites in the southern Laos provinces of Savannakhet, Salavan, Sekong, Champasak, and Attapeu. Samples were collected from symptomatic patients of all ages who had been confirmed positive for *P falciparum*, using standard procedures of the GenRe-Mekong genetic surveillance project.^18^ Samples from Attapeu collected by the TRAC study in the 2011–12 malaria season were also included; the methods for collection of these samples have been described previously.^3^ No additional inclusion and exclusion criteria were applied to the selection of the patients. No additional inclusion or exclusion criteria were applied to the selection of dried blood spot samples. Patients signed a written consent form allowing the collection of blood spots for further analysis. For ancestry analyses, we used whole-genome sequencing (WGS) data from parasites included in the public release (Feb 24, 2021) of the MalariaGEN *P falciparum* Community Project v6.0,^19^ as well as WGS data from Laos samples from GenRe-Mekong and genotyped in subsequent partner releases (appendix 2 p 5). Ethical approvals were obtained from the National Ethics Committee for Health Research of the Health Ministry of the Lao People's Democratic Republic and the Oxford Tropical Research Ethics Committee. A full description of the methods is shown in appendix 2 (pp 12–14).

### Genotyping and WGS

Parasite genomic DNA was extracted from the dried blood spot sample, amplified, and processed on MiSeq sequencers (Illumina, San Diego, CA, USA) using the SpotMalaria v2 amplicon sequencing and genotyping platform. The genotyping process produced a standardised set of variants and haplotypes relevant to drug resistance, as well as a 101-variant genetic barcode. The genetic barcode provides a genome-wide summary of a sample's genetic variants and is used when comparing samples in epidemiological analyses.^18^ We filtered genetic barcodes to reduce errors due to data missingness, by removing barcode variants with more than 25% of missing calls, and samples with more than 25% of missing calls in the remaining variants.

For WGS samples, genotypes were called from Illumina short reads at 1 042 396 quality-filtered nuclear biallelic single-nucleotide polymorphisms (SNPs) using the standardised MalariaGEN *P falciparum* Community Project genotyping pipeline v6.0.^19^ Copy number estimations for the *plasmepsin2/3* (*pm23*) or *multidrug resistance 1* (*mdr1*) genes were performed by detection of a duplication breakpoint^19^ or by quantitative PCR estimation.^20^

### Population structure analyses

Pairwise genetic distances (a measure of sample differentiation) were computed from genetic barcodes. Heatmaps of pairwise distance matrices were produced using the ComplexHeatmap R package (version 2.8.0).^21^ The same matrices were used as input to principal coordinate analysis, a technique that allows the grouping of samples by similarity.

Samples collected in Attapeu between Jan 1, 2011, and April 1, 2021, were clustered by creating a graph based on barcode genetic distances and applying a community detection algorithm to partition the sample set. The same method was used to cluster 9609 additional samples collected from eight countries by GenRe-Mekong.^18^ Spatial distribution of clusters was visualised using bespoke R code and map data from Natural Earth.

Population-level genetic diversity was assessed by the mean expected heterozygosity across all barcode SNPs, computed from allele frequency estimates after removing missing allele calls.

### Statistical analysis

All statistical analyses were performed using R (version 4.2.0). For pairwise comparison between groups we used Wilcoxon rank-sum tests with a method to adjust the p value to control for false discovery rate, using the function pairwise.wilcox.test(x, p.adjust.method=“fdr”). For comparison between multiple populations, we used the kruskal.test function to perform Kruskal-Wallis tests. p<0·05 was considered statistically significant. Associations of *kelch13* genotypes with the outbreak in Attapeu were tested by a logistic regression model.

### Ancestry analysis

Pairwise identity by descent (IBD) analysis was performed on genotypes with 53 150 SNPs filtered by variability using hmmIBD.^22^ Visualisations of recombination patterns were produced by bespoke R scripts, which are available from the authors on request.

### Role of the funding source

The funders of the study had no role in study design, data collection, data analysis, data interpretation, or writing of the report.

## Results

**Figure 1:**
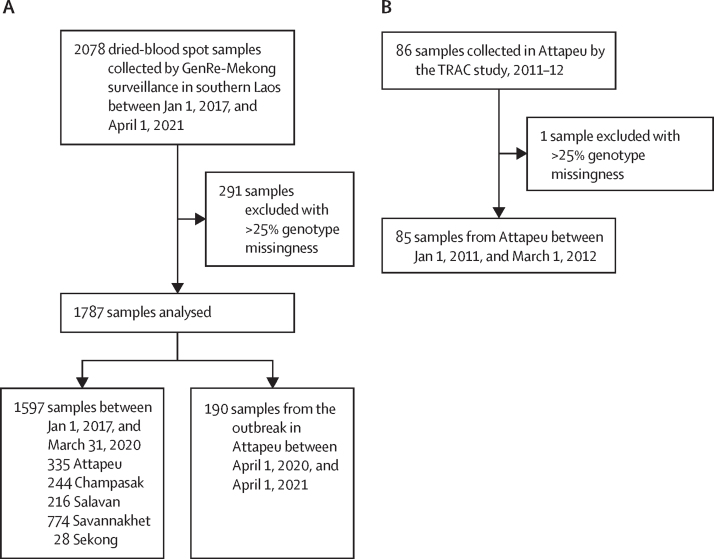
Sample selection The diagram shows how the analysed sample set was derived from Laos samples contributed by GenRe-Mekong (A) and by the earlier TRAC study (B), and the composition of this sample set by province and time period.

The southern Laos province of Attapeu had a 130% increase in case numbers during the 2020–21 malaria season (378 cases in 2019 and 868 cases in 2020), in contrast with a low *P falciparum* incidence in other endemic provinces, including Savannakhet where the highest proportion of cases had been seen in previous years (2017–19). These changes in case numbers were reflected by the number of dried blood spot samples collected by GenRe-Mekong (appendix 2 p 6). To investigate this outbreak, we analysed genotypes from 2078 dried blood spot samples collected by the GenRe-Mekong project at public health facilities in southern Laos, which included 249 samples collected from cases in Attapeu Province during the outbreak, between April 1, 2020, and April 1, 2021, before COVID-19 pandemic lockdowns came into effect. These samples were compared to the remaining 1829 samples, collected by routine surveillance in five provinces (including 622 in Attapeu Province) between Jan 1, 2017, and March 31, 2020; and to 86 samples from Attapeu, collected by the TRAC study between Jan 1, 2011, and March 1, 2012.^3^ To support analyses of diversity and genetic distance, we analysed genetic barcodes comprising 101 SNPs selected for their variability and low mutual linkage.^18^ We excluded samples with high genotype missingness (>25%), resulting in a final set of 1787 samples, including 190 collected during the outbreak in Attapeu (figure 1; appendix 2 p 3).

**Figure 2:**
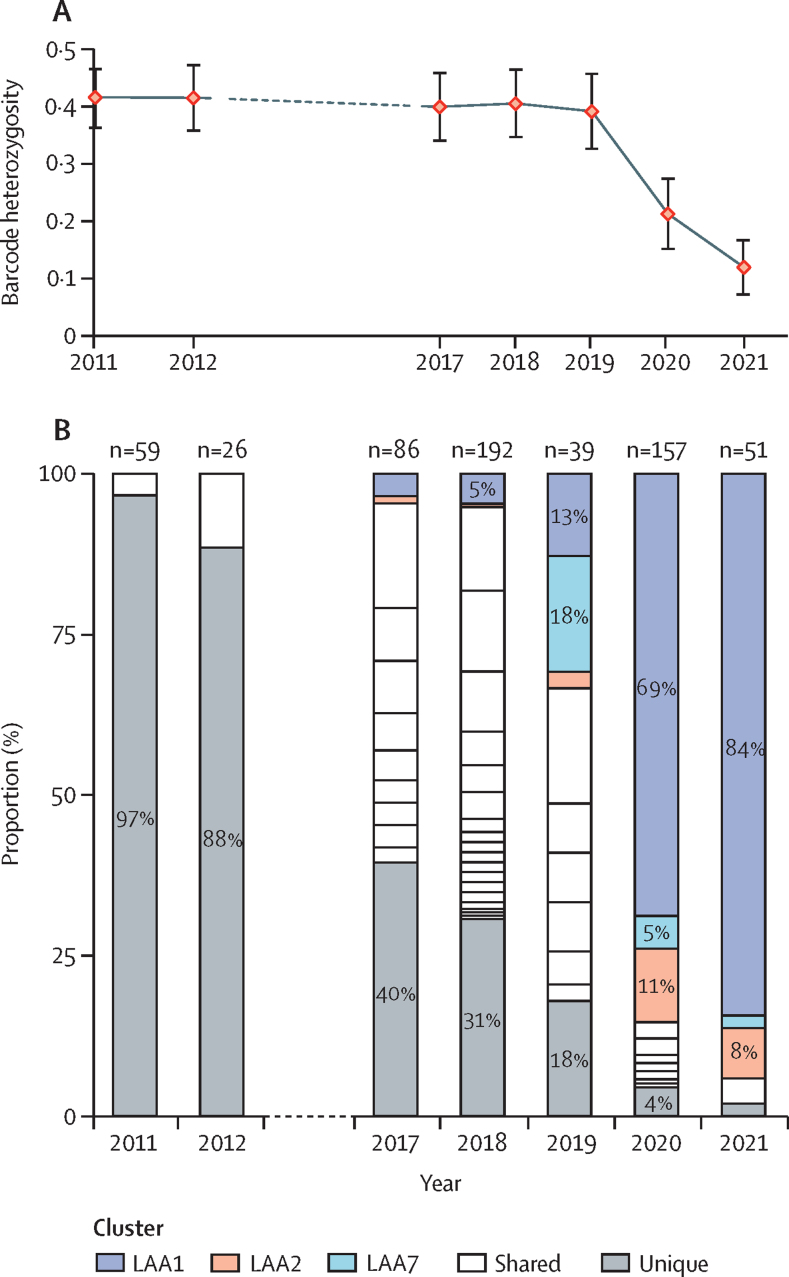
Temporal changes in the population diversity in Attapeu province (A) Changes in population diversity over time. Mean single-nucleotide polymorphism heterozygosity across all genetic barcode loci is estimated as a measure of diversity for each year. The dashed line indicates a 5-year gap where data is not available. Error bars show standard deviation. (B) Increasing prevalence of clusters with highly related parasites (≥95% identity) and decreasing prevalence of parasites with unique barcodes (not belonging to a cluster). Bar segments represent the clusters identified each year, their size being proportional to the number of cluster members; parasites with unique barcodes are grouped in a grey segment at the bottom of the bar. Clusters that dominate the population during the 2020–21 outbreak (LAA1, LAA2, and LAA7) are shown in colour. The total number of samples in each year is shown.

As a first step, we compared the diversity of genetic barcodes in outbreak parasites with that of parasites circulating in Attapeu in previous years, using the average SNP heterozygosity across all barcoding loci as a measure. In the 2017–19 period, diversity remained stable, at similar levels to those in 2011–12, with mean heterozygosity in the range of 0·39 (SD 0.11) to 0·42 (0·13). Starting in 2020, however, diversity collapsed, with heterozygosity reaching 0·12 (0·13) in 2021 (p<0·0001 when comparing 2020–21 *vs* 2012–19; figure 2A). Before the outbreak, diversity in other provinces was lower (range of 0·36 [0∙11] to 0·38 [0∙14]) than in Attapeu (0·41 [SD 0·11]; p=0·040) but significantly higher than in the most recent Attapeu samples (p<0·0001; appendix 2 p 7). We can conclude that the outbreak was characterised by a massive loss of diversity, unprecedented for Attapeu and for Laos in general. It is worth noting that Savannakhet Province had seasonal case number rises in three previous seasons (appendix 2 p 6), but these epidemics were not accompanied by a big reduction in diversity, suggesting that their drivers differed in nature from those of the Attapeu outbreak.

Expecting the reduction in diversity to be caused by expanding populations, we sought to investigate the population structure changes that accompanied the outbreak. To identify populations of highly related individuals, we estimated pairwise genetic distances and used these to cluster parasites with at least 95% barcode identity. This analysis identified 30 clusters with more than two members within the Attapeu sample set. In 2011–12, before the multidrug-resistant KEL1/PLA1 parasite migrated into the southernmost provinces of Laos,^18^, ^23^ genetic barcode diversity was very high, such that at least 88% of parasites did not cluster with other parasites (57 [97%] of 59 in 2011 and 23 [88%] of 26 in 2012; figure 2B). In 2017–18, considerably more population structure emerged, as parasites formed several clusters, and the proportion of parasites carrying unique barcodes dropped to 40% (34/86) in 2017 and 31% (59/192) in 2018. Several of these clusters might represent sub-strains of KEL1/PLA1, as previously described.^23^ Starting in 2019 and leading into the outbreak period, KEL1/PLA1 clusters disappeared and three clusters (labelled LAA1, LAA2, and LAA7) grew to dominate the population. Some members of these clusters were circulating at low frequency before 2019, but subsequently expanded so aggressively that they constituted over 94% (48/51) of samples in 2021. The most rapidly expanding cluster (LAA1) rose to 84% (43/51) prevalence in 2021, and was clearly responsible for most of the diversity reduction. The second largest cluster (LAA2) expanded more gradually, reaching a frequency of 8% (4/51) in 2021, and the third cluster (LAA7) appeared to shrink in size as LAA1 expanded. To summarise, *P falciparum* underwent a progressive increase in population structure in Attapeu, likely to have been driven initially by imported artemisinin-resistant strains. However, in the 2019–21 period, a small number of clonally expanding parasite clusters effectively replaced the previous populations, strongly suggesting that the outbreak resulted from a selective sweep.

**Figure 3:**
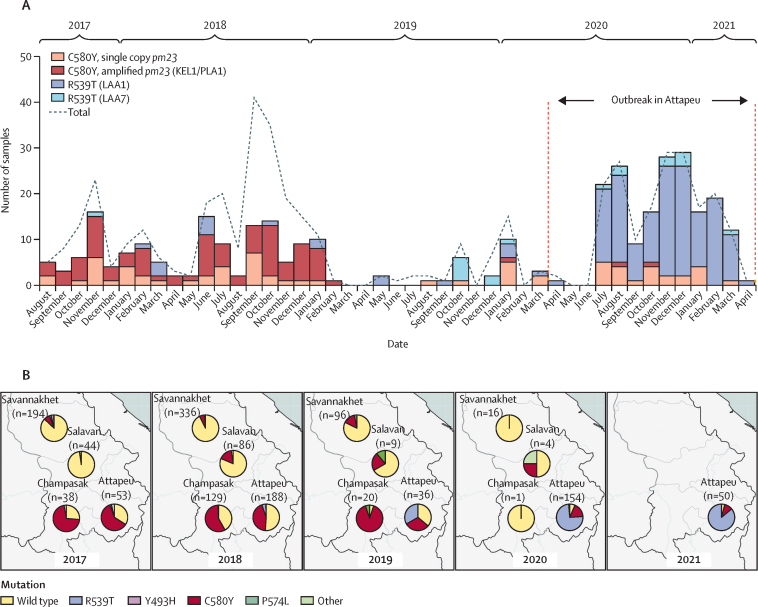
Prevalence of *kelch13* mutations (A) Temporal distribution of selected *kelch13* mutations in Attapeu province, for each month from August, 2017, to April, 2021. The bars show the counts for: C580Y mutants without *pm23* amplification (orange), C580Y mutants with *pm23* amplification (KEL1/PLA1; dark red), LAA1 R539T mutants (dark blue), and LAA7 R539T mutants (light blue). A dashed line shows the total number of samples collected in the same periods (ie, including samples with wild-type *kelch13* and others). (B) Spatiotemporal distribution of *kelch13* mutations in four provinces of southern Laos (Attapeu, Champasak, Salavan, and Savannakhet). Number of samples in each province is shown. Sekong Province was not included due to low number of samples. *pm23*=*plasmepsin2/3*.

To identify possible selection drivers, we characterised clusters according to the genotypes at key drug resistance markers (appendix 2 pp 3–4). As expected, in 2017–18, nine clusters comprised multidrug-resistant parasites likely to be variants of the KEL1/PLA1 strain, as they carried the *kelch13* Cys580Tyr (C580Y) mutation (a marker of resistance to artemisinin) and the *pm23* amplification (a marker of resistance to piperaquine).^23^, ^24^ KEL1/PLA1 proliferated in several Greater Mekong Subregion countries where dihydroartemisinin–piperaquine was the front-line ACT, and is known to have penetrated Laos, although dihydroartemisinin–piperaquine was not used in Laos.^18^ During the outbreak, KEL1/PLA1 rapidly disappeared within a year and was replaced by the three outbreak clusters (appendix 2 p 8), all carrying alleles associated with resistance to chloroquine, sulfadoxine, pyrimethamine, and artemisinin (appendix 2 pp 3–4). LAA2 parasites carried the C580Y mutation, although the absence of *pm23* amplifications suggests that they were not closely related to KEL1/PLA1 parasites. The LAA1 and LAA7 clusters both comprised parasites carrying the *kelch13* Arg539Thr (R539T) mutation, without *pm23* amplification. Therefore, a rapid switch in prevalence of *kelch13* mutations occurred in 2020, with R539T mutants prevailing over those carrying C580Y, causing the excess of cases (figure 3A). We found the R539T mutation to be very strongly associated with the outbreak onset (p<0·0001; appendix 2 p 5). Because LAA1 and LAA7 parasites were only found in Attapeu Province (appendix 2 pp 8–9), it is probable that these lineages have emerged locally. The R539T mutation was not detected in other provinces of Laos (figure 3B) but was present at low frequency in Attapeu before the outbreak. Most (48 [87%] of 55) of the LAA1 parasites lacked *mdr1* amplification, a molecular marker associated with mefloquine resistance.^25^ All LAA2 and LAA7 carry a single copy of *md1*, predicted to be sensitive to the partner drug mefloquine.

**Figure 4:**
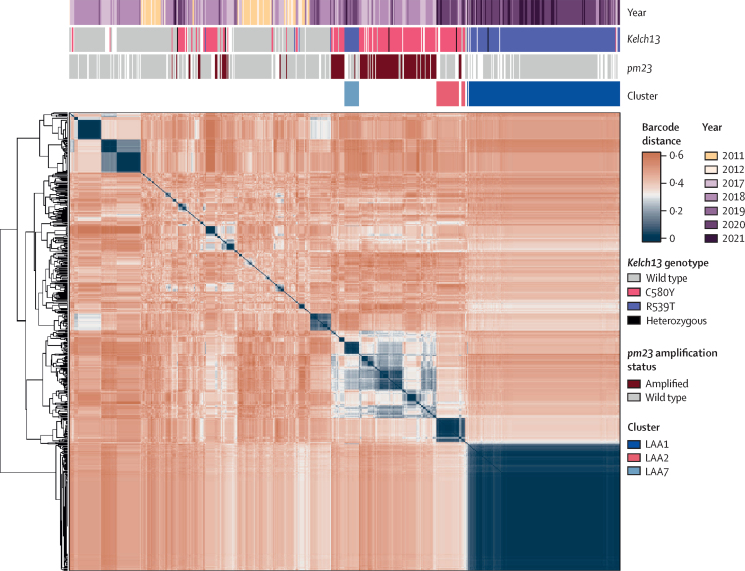
Heatmap of relatedness between populations in Attapeu based on pairwise genetic barcode distances A pairwise distance matrix was calculated from a set of 610 samples from Attapeu, using 96 quality-filtered barcode single-nucleotide polymorphisms. The arrangement of samples on the heatmap is determined by hierarchical clustering; the dendrogram on the left shows the clustering hierarchy. Colour bars at the top of the heatmap indicate for each sample: the year of sampling; the *kelch13* genotype; the *pm23* amplification status; and the cluster to which the sample belongs. Heterozygous means a mixed sample containing parasites carrying more than one *kelch13* genotype. Heatmap cell colours reflect the pairwise distances between samples: shades of blue represent high pairwise similarity, the darkest blue corresponding to identical genetic barcodes; shades of red signify low barcode similarity, with deepest red representing a distance of 0·6 (ie, 60% of the barcode alleles differ between a pair of samples). *pm23*=*plasmepsin2/3*.

To better understand the relationship between parasite clusters, we constructed a heatmap of genetic distances between pairs of individuals (figure 4). The most prominent feature on the heatmap is the extremely high degree of similarity among members of the LAA1 cluster (median distance=0·0 [IQR 0·016]), showing very little evidence of recombination between this essentially clonal group and other clusters. LAA1 also appear to diverge from other Attapeu parasites (median pairwise distance between LAA1 and non-LAA1 parasites=0·42 [IQR 0·030]; p<0·0001). Although clonality and differentiation are typical features of founder populations, LAA1 parasites circulated in small numbers for several seasons before their expansion, supporting the notion that the expansion is the result of a selective sweep.

The characteristics of LAA1 can be contrasted with those of KEL1/PLA1 clusters, which form a fragmented group of related clusters in the heatmap, consistent with differentiated sub-lineages. LAA7 is also located within this group, although it carries an R539T allele rather than C580Y, and does not possess the *pm23* amplification (two key features of KEL1/PLA1). This finding suggests that LAA7 might have originated from KEL1/PLA1 parasites and, through recombination, acquired a different *kelch13* variant and lost the *pm23* amplification. The LAA2 cluster, in contrast, carries the C580Y allele but does not group with the KEL1/PLA1 clusters, suggesting that it derives from a separate C580Y lineage. Additional analyses using principal coordinates analysis corroborated these observations, showing that LAA1 sets itself apart from other clusters; that LAA2 is distinct from earlier KEL1/PLA1 clusters; and that LAA7 is more closely related to the C580Y parasites than to LAA1 (appendix 2 p 10).

These findings raise questions about the origins of the outbreak clusters, particularly those carrying the *kelch13* R539T mutation. Did they emerge because of independent mutation events, or are they descendants of previously identified R539T strains?^15^, ^26^, ^27^ Can recombination events explain the similarities between LAA7 and KEL1/PLA1? To answer these questions, we reconstructed the ancestry of the outbreak strains using IBD analyses, which identify genome stretches in a sample pair that are statistically likely to have inherited from a common ancestor. We used WGS data from Laos and its neighbouring countries, contributed to the MalariaGEN *P falciparum* Community Project v6.4, including a small number of LAA1, LAA2, and LAA7 samples collected by the GenRe-Mekong surveillance project (appendix 2 p 5).

**Figure 5:**
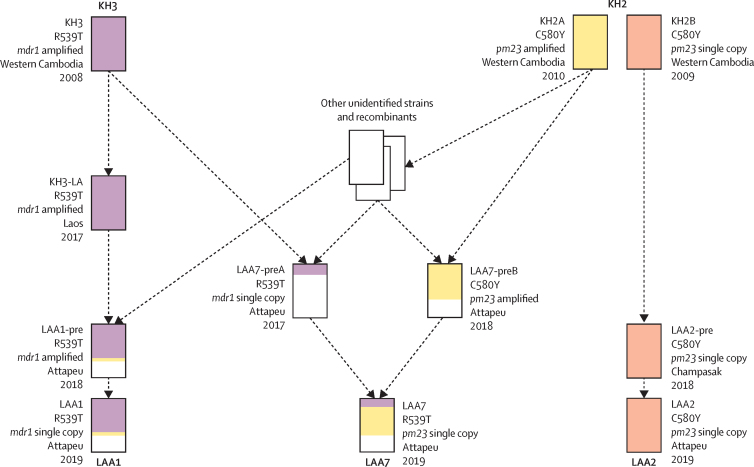
Reconstruction of ancestry of the Attapeu outbreak strains The diagram shows the relationship between strains analysed for identity by descent (appendix 2 p 5). Each rectangle represents a sequenced sample: the three samples at the top represent older *kelch13* mutants from Cambodia, the three samples at the bottom represent the Attapeu outbreak clusters; the remaining rectangles represent intermediate descendants and other parasite strains. Each rectangle is filled with coloured segments that show the proportion of contribution from the older Cambodian strains; white segments represent contributions from unknown sources. Next to each rectangle, we indicate: the sample label, the *kelch13* mutation and the amplification status of *pm23* or *mdr1*, and the place and year of sampling. Dashed arrows suggest putative inheritance paths, derived from observing identity by descent patterns, but might not denote an actual direct descendance of one sample from another (as opposed to both having common ancestry). Furthermore, numerous intermediate ancestry steps are not shown in this highly simplified depiction. *mdr1*=*multidrug resistance 1. pm23*=*plasmepsin2/3*.

We considered some of the oldest *kelch13* mutants available, particularly the early founder populations circulating in western Cambodia in 2008 and 2009. Two of these populations, labelled KH2 and KH3 in previous work,^15^ were found to harbour C580Y and R539T mutants respectively.^27^ When comparing LAA1 with one of the R539T mutants (KH3, collected in western Cambodia in 2008), we found 58·8% IBD, including a region containing the *kelch13* gene (appendix 2 p 11; figure 5). This finding indicates that the artemisinin resistance variant carried by LAA1 did not emerge independently in Laos, but was inherited from strains circulating over a decade ago. The large IBD stretches observed indicate limited linkage breakdown, implying that very few recombination events occurred during that time. This hypothesis was confirmed by an analysis of R539T mutants circulating in Laos in 2017 (KH3-LA), which were nearly identical to Cambodian KH3 (IBD around 100%). Hence, this R539T lineage circulated for almost a decade essentially unchanged, spreading across national borders, before contributing to recombination events that produced LAA1, probably around 2017 when the earliest LAA1 members were sampled. Although no donor was identified for the remainder of the LAA1 genome, a *kelch13* wild-type parasite from Champasak (LAA1-preWT) had 27·1% IBD with LAA1, mostly complementary to the KH3 contributions, suggesting that the recombination events that produced LAA1 involved local wild-type parasites. In contrast, a 2009 Cambodian KH2 C580Y mutant (KH2A) only shared 8·3% IBD with LAA1 ancestry, partly overlapping with the KH3 contribution (appendix 2 p 11).

The ancestry of LAA2 was simpler to reconstruct: its genome is essentially identical to that of the KH2A parasite (IBD >99%), suggesting that LAA2 expanded from a strain that has spread regionally, since 2009 or earlier, probably from western Cambodia, approximately 500 km from Attapeu. These parasites carry the C580Y mutation, but not the *pm23* amplification which characterises KEL1/PLA1, as confirmed by the IBD levels (36·9%) when comparing LAA2 with an early Cambodian C580Y mutant carrying the amplification (KH2B, collected in 2010), suggesting that LAA2 derived from a lineage related to but distinct from KEL1/PLA1.

IBD analyses showed that the LAA7 cluster probably originated from the recent recombination of two local strains, an R539T mutant (LAA7-preA, Attapeu, 2017), which contributed 49·0%, and a C580Y mutant (LAA7-preB, Attapeu, 2018), which contributed 45·1% (appendix 2 p 11). The two donor strains are themselves recombinants showing substantial IBD with early Cambodian mutants: LAA7-preA shares 23·1% of its genome with KH3, including the R539T *kelch13* variant region, eventually inherited by LAA7, and LAA7-preB appears to be a KEL1/PLA1 strain closely related to earlier Cambodian strains (65·5% IBD with KH2B). The contribution of LAA7-preB has resulted in a high level of IBD between LAA7 and KEL1/PLA1, explaining the relationship observed in the genetic distance heatmap.

## Discussion

In regions approaching malaria elimination, outbreaks threaten progress and can result in disease resurgence. We have shown that genetic surveillance offers informative new perspectives on outbreaks, by studying three key aspects: genetic diversity at population level, markers of drug resistance, and ancestry of the outbreak strains.

The study of genetic diversity showed that the case number increase in the Laos outbreak was primarily caused by the clonal expansion of the LAA1 multidrug-resistant strain, carrying the *kelch13* R539T mutation. This expansion occurred very rapidly, effectively replacing the dominant KEL1/PLA1 population in a single season. KEL1/PLA1 strains had become established in Champasak and Attapeu Provinces, probably imported from neighbouring countries where dihydroartemisinin–piperaquine was the ACT of choice. Although LAA1 parasites were present since 2018, they remained at low frequency while KEL1/PLA1 dominated. The KEL1/PLA1 population in Attapeu collapsed in 2019–20, coinciding with the end of dihydroartemisinin–piperaquine usage in neighbouring northeast Thailand and Viet Nam, soon after Cambodia ceased use of this treatment. These policy changes most probably reduced selective pressures on the parasite population, and it is also possible that movement restrictions in 2020, due to the COVID-19 pandemic, reduced the number of imported parasites. The demise of KEL1/PLA1 left a vacuum that was rapidly filled by the rise in frequency of LAA1.

The mainly clonal nature of the LAA1 expansion, with little evidence of interbreeding, is characteristic of populations under strong selective pressure. In outbreaks driven by increased transmission due to environmental or human factors, higher levels of recombination and greater diversity would be expected. The very low diversity seen in this outbreak was unusual when compared both with other provinces of Laos, and to previous seasons in Attapeu. It is improbable that the scarcity of parasite variety was a limiting factor, because we observed two additional clusters and some wild-type parasites circulating during the outbreak. Such low levels of recombination could occur if LAA1's selective advantages were conferred by a set of variants that would be disrupted through recombination with different genetic backgrounds, so that offspring from selfing (mating with genetically identical individuals) would retain the selective advantage and have a greater chance of survival than recombinants. This scenario could also explain how the LAA2 strain preserved its genome unchanged for at least 12 years since it was first sampled in Cambodia. This lineage appears to have retained its competitiveness through selfing for more than a decade and had a revival hundreds of km from its place of origin. This is not a unique case: essentially identical KH3 parasites were sampled in different countries, almost a decade apart, before their contribution to the LAA1 genome. Overall, IBD patterns indicate that successful recombinants of these multidrug-resistant strains are infrequent, as evidenced by the limited linkage breakdown in the LAA1 and LAA7 genomes.

All genetic evidence points to a selective sweep being the cause of the Attapeu outbreak. Additionally, we note that the KEL1/PLA1 population also waned in neighbouring Champasak, but only Attapeu had a surge in case numbers, which further suggests that the LAA1 population, local to this province, had a selective advantage. Unfortunately, our data cannot pinpoint the exact nature of this selective advantage. Selection can be driven by any factor that increases a parasite's chance to transmit—eg, increased gametocyte production or adaptation to vectors. However, given the recent history of malaria in the Greater Mekong Subregion, antimalarial drug resistance seems the most probable driver; hypothetically, LAA1 parasites might have reduced sensitivity to lumefantrine, the front-line ACT partner drug in Laos. This hypothesis cannot be confirmed by our data because there is no widely accepted marker of lumefantrine resistance; LAA1 parasites mostly do not have the *mdr1* gene amplification, a proposed candidate marker,^25^ which was a feature of their R539T mutant precursors. Still, it is possible that LAA1 inherited unknown drug resistant mutations from unknown contributors. We strongly recommend that artemether–lumefantrine efficacy be confirmed in Attapeu Province, where it would also be valuable to test the in vitro response to partner drugs. In response to the outbreak, the Centre of Malariology, Parasitology, and Entomology of Laos has conducted active case detection in the community at high risk and started a therapeutic efficacy study of artemether–lumefantrine in Attapeu, Savannakhet, and Sekong.

Our IBD analysis indicates that novel artemisinin-resistant lineages have inherited *kelch13* haplotypes from early mutants circulating in western Cambodia. In other words, the *kelch13* mutations circulating in the eastern Greater Mekong Subregion originate from a small number of mutation events,^27^ in contrast with the many independently emerged mutations observed in Myanmar.^28^, ^29^ Perhaps, the small number of founder populations described a decade ago^15^ had already undergone a selection process that weeded out less fit mutants, and these populations and their derivatives have been very resilient over time. Even while KEL1/PLA1 parasites seemed to head for complete domination,^30^ R539T parasites and C580Y parasites without *pm23* amplification remained in circulation. This alternation in prevalence was probably triggered by human behaviours affecting selective pressures, such as changes in the choice of front-line treatment. Therefore, it remains crucial to monitor all *kelch13* mutant varieties in this region, rather than focus on variants whose dominance might be temporary. Using WGS data, drug resistant strains can be systematically catalogued, and tracked by genetic markers. Here, we have shown that ancestry reconstruction methods can be powerful tools for this type of investigation.

As elimination approaches, public health interventions will apply increasingly stronger selective pressures on the parasite population, which might lead to new emerging forms of drug resistance, rapid sweeps, and unexpected outbreaks and resurgence. To maintain ACT efficacy, it will be crucial for national malaria control programmes to be empowered with an arsenal of tools with which to respond to these phenomena. Routine genetic surveillance, which is relatively straightforward and affordable to implement,^18^ can address important use cases that go beyond the simple monitoring of drug resistance markers; here, we have shown it is a powerful tool for analysing the causes and dynamics of outbreaks. Although the development of a generalised methodology to address this use case will require comparative analyses of other outbreaks, which are outside the scope of this work, we believe that the present work provides the essential templates for future work. Using simple 101-SNP barcodes, we identified and characterised three different expanding strains and showed an abnormal drop in diversity, indicative of a selective sweep. Crucially, rapid processing enabled us to communicate findings to the National Malaria Control Programme before the beginning of the following season, so that they could plan their response. The addition of WGS data enabled a study of the origins of the outbreak populations, and their relationship to well known resistant strains and local parasites. There is clear value in analysing genomic epidemiology at different levels of resolution, using simpler techniques to answer the pressing questions rapidly, and studying the underlying phenomena and processes using more detailed methods. We encourage the genomic epidemiology and surveillance communities to continue contributing to shared genomic datasets that support tackling these and other important public health use cases.

## Data sharing

This publication uses data from the MalariaGEN *P falciparum* Community Project.^19^ Genetic Report Card data for the 2164 samples from Laos used in the present analyses are provided in appendix 3, which contains a data dictionary sheet (README) that details the fields included.

## Declaration of interests

We declare no competing interests.

## Supplementary Materials

Lao translation of the abstract
