# Supplementary material for: Malaria outbreak in Laos driven by a selective sweep for Plasmodium falciparum kelch13 R539T mutants: a genetic epidemiology analysis
Source: Lancet Infect Dis. 2023 May;23(5):568–77. doi: 10.1016/S1473-3099(22)00697-1 (PMC10914674; doi:10.1016/S1473-3099(22)00697-1)
Supplement: Lao translation of the abstract [file mmc1.pdf]

THE LANCET
Infectious Diseases

Supplementary appendix 1

This translation in Lao was submitted by the authors and we reproduce it as supplied. It has not been peer reviewed. *The Lancet*'s editorial processes have only been applied to the original in English, which should serve as reference for this manuscript.

*“ການແປເປັນພາສາລາວນີ້ ແມ່ນ ຖືກສົ່ງມາຈາກຄະນະຜູ້ຂຽນ ແລະ
ພວກເຮົາພຽງແຕ່ຈັດພິມຕາມທີ່ໄດ້ຈັດສົ່ງມາ. ໂດຍບໍ່ໄດ້ຖືກທົບທວນຄືນໂດຍລະອຽດ.
ຂັ້ນຕອນທາງບັນນາທິການຈາກລານເຊັດ ແມ່ນ ໄດ້ຖືກຮັບຮອງຕາມຕົ້ນສະບັບທີ່ເປັນພາສາອັງກິດ
ເຊິ່ງຄວນຖືເປັນບ່ອນອີງສຳລັບບົດນີພິນສະບັບນີ້.”*

Supplement to: Wasakul V, Disratthakit A, Mayxay M, et al. Malaria outbreak in Laos driven by a selective sweep for Plasmodium falciparum kelch13 R539T mutants: a genetic epidemiology analysis. *Lancet Infect Dis* 2022; published online Nov 30. https://doi.org/10.1016/S1473-3099(22)00697-1.

ບົດຄັດຫຍໍ້

ຄວາມເປັນມາ

ການລະບາດຂອງໄຂ້ມາລາເຣຍ ສ້າງບັນຫາ ແລະ ຄວາມກັງວົນ ດ້ານສາທາລະນະສຸກທີ່ສຳຄັນ ເຊິ່ງໄດ້ເຮັດໃຫ້ຫລາຍໆຂົງເຂດທີ່ກຳລັງຈະລົບລ້າງພະຍາດດັ່ງກ່າວ ໄດ້ກັບຄືນມາເປັນພື້ນທີ່ລະບາດບົ່ມຊ້ອນຂອງພະຍາດຕໍ່ໄປອີກ. ພວກເຮົາໄດ້ເຮັດການສຶກສາ ຫາຕົ້ນເຫດການລະບາດຂອງໄຂ້ມາລາເຣຍຊະນິດ ປ. ຟານຊິປາຣອມ ທີ່ແຂວງອັດຕະປື, ສປປ ລາວ ໃນປີ 2020-2021 ໂດຍນຳໃຊ້ວິທີລະບາດວິທະຍາທາງພັນທຸກຳ ເພື່ອສຶກສາລົງເລິກດ້ານການເຄື່ອນເໜັງຂອງປະຊາກອນເຊື້ອກາຝາກ ແລະ ສາເຫດການລະບາດ.

ວິທີວິທະຍາການຄົ້ນຄວ້າ

ພວກເຮົາໄດ້ສຶກສາລະຫັດທາງພັນທຸກຳຂອງເຊື້ອ ປ. ຟານຊິປາຣອມ ຈຳນວນ 2,164 ຕົວຢ່າງ ທີ່ໄດ້ຈາກພາກໃຕ້ຂອງ ສປປ ລາວ ໃນຊ່ວງ ວັນທີ 1 ມັງກອນ 2017 ຫາວັນທີ 1 ເມສາ 2021, ໃນນີ້ 249 ຕົວຢ່າງ ໄດ້ຈາກແຂວງອັດຕະປື ໃນຊ່ວງທີ່ມີການລະບາດ ລະຫວ່າງວັນທີ 1 ເມສາ 2020 ຫາວັນທີ 1 ເມສາ 2021 ດ້ວຍການເຝົ້າລະວັງທີ່ເຮັດເປັນປະຈຳ. ພວກເຮົາໄດ້ນຳໃຊ້ລະຫັດທາງພັນທຸກຳຈາກຕົວຢ່າງເຊື້ອດັ່ງກ່າວ ເພື່ອສຶກສາເບິ່ງລັກສະນະການປ່ຽນແປງທາງລະບາດວິທະຍາ ເຊິ່ງອາດເປັນຊ່ອງທາງໄຂປິດສະໜາຂອງການລະບາດຄັ້ງນີ້, ແລະ ໄດ້ສຶກສາເບິ່ງລັກສະນະຄວາມຫລາກຫລາຍ ແລະ ໂຄງສ້າງຂອງປະຊາກອນເຊື້ອກາຝາກ. ນອກນີ້ ຍັງໄດ້ນຳໃຊ້ຂໍ້ມູນລະຫັດທາງພັນທຸກຳຂອງເຊື້ອ ທີ່ເຄີຍເກັບກຳມາກ່ອນໜ້ານີ້ ເພື່ອໃຊ້ເປັນບ່ອນອີງໃນການສົມທຽບກັບເຊື້ອສາຍພັນທີ່ມີການລະບາດ ໂດຍນຳໃຊ້ການວິເຄາະແບບ IBD (Identity-by-descent).

ຜົນການສຶກສາຄົ້ນຄວ້າ

ພວກເຮົາພົບວ່າ: ປະຊາກອນເຊື້ອກາຝາກໄຂ້ມາລາເຣຍທີ່ເກີດການລະບາດນັ້ນ ມີການສູນເສຍຄວາມຫລາກຫລາຍທາງພັນທຸກຳ ທີ່ບໍ່ເຄີຍມີມາກ່ອນ ເຊິ່ງຕົ້ນຕໍແມ່ນມີສາເຫດຈາກການແຜ່ກະຈາຍຢ່າງໄວວາ ຂອງເຊື້ອສາຍພັນທີ່ຕ້ານຕໍ່ຢາປິ່ນປົວ (LAA1) ເຊິ່ງເຊື້ອສາຍພັນນີ້ມີການກາຍພັນຂອງຢີນ *kelch13* R539T. ເຊື້ອສາຍພັນ LAA1 ດັ່ງກ່າວໄດ້ເຂົ້າມາແທນທີ່ສາຍພັນກ່ອນ ເຊິ່ງແມ່ນສາຍພັນ *kelch13* Cys580Tyr (C580Y) mutants ທີ່ມີການຕ້ານຕໍ່ຢາ dihydro-artemisinin-piperaquine (KEL1/PLA1). ສາຍພັນ LAA1 ນີ້ ມີການສືບເຊື້ອສາຍທາງພັນທຸກຳປະມານ 58.8% ຂອງສາຍພັນທີ່ພົບໃນປະເທດກຳປູເຈຍ ໃນປີ 2008 ທີ່ຜ່ານມາ. ນອກນີ້ ຍັງພົບສາຍພັນທີສອງ ທີ່ມີການລະບາດ (LAA2) ເຊິ່ງມີການກາຍພັນຂອງຢີນ *kelch13* C580Y ແລະ ມີລັກສະນະທາງພັນທຸກຳຄ້າຍຄຽງກັບເຊື້ອສາຍພັນທີ່ພົບໃນປະເທດກຳປູເຈຍໃນປີ 2009. ສາຍພັນທີສາມທີ່ພົບເຫັນໃນປະລິມານທີ່ຕໍ່າ ໄດ້ແກ່ສາຍພັນ LAA7 ເຊິ່ງເກີດຈາກການປະສົມລະຫວ່າງເຊື້ອສາຍພັນ KEL1/PLA1 ກັບສາຍພັນ a *kelch13* R539T.

ການແປຜົນ

ຜົນຈາກການສຶກສາຄັ້ງນີ້ ສະແດງໃຫ້ເຫັນຢ່າງຊັດເຈນວ່າ: ການເກີດລະບາດດັ່ງກ່າວ ແມ່ນເກີດຈາກການເລືອກເຟັ້ນສະເພາະຂອງເຊື້ອສາຍພັນ (selective sweep) ທີ່ມີການຕ້ານຕໍ່ຢາປິ່ນປົວຫລາຍຊະນິດ. ປະຊາກອນເຊື້ອກາຝາກທີ່ຕ້ານຕໍ່ຢາປິ່ນປົວ ອາດໂຄຈອນໃນພື້ນທີ່ໃນລະດັບອັດຕາທີ່ຕໍ່າເປັນເວລາຫລາຍໆປີ ກ່ອນທີ່ພວກມັນຈະກາຍເປັນກຸ່ມສາຍພັນທີ່ໃຫ່ຍຂຶ້ນຢ່າງຫລວງຫລາຍ ແລະ ເພີ່ມຂຶ້ນໃນທັນທີທັນໃດ ພາຍຫລັງທີ່ເງື່ອນໄຂການເລືອກເຟັ້ນສະເພາະພວກມັນອຳນວຍໃຫ້ ເຊັ່ນຕົວຢ່າງ ເມື່ອມີການປ່ຽນແປງແນວທາງການປິ່ນປົວດ້ວຍຢາຊະນິດໃໝ່ ເປັນຕົ້ນ. ສະນັ້ນ ການເຝົ້າລະວັງທາງພັນທຸກຳຂອງເຊື້ອກາຝາກໄຂ້ມາລາເຣຍ ຈະຊ່ວຍຍຸດທະສາດໃນການລົບລ້າງພະຍາດດັ່ງກ່າວ ດ້ວຍການສຶກສາຄຸນລັກສະນະສະເພາະຕ່າງໆຂອງການລະບາດ ເຊັ່ນ: ການສຶກສາຄວາມຫລາກຫລາຍຂອງປະຊາກອນເຊື້ອກາຝາກ, ອັດຕາຊຸກຊຸມຂອງຢີນທີ່ບົ່ງບອກເຖິງການຕ້ານຕໍ່ຢາປິ່ນປົວ, ແລະ ຕົ້ນກຳເນີດຂອງເຊື້ອສາຍພັນທີ່ກໍ່ໃຫ້ເກີດການລະບາດ.
