# Supplementary material for: Malaria outbreak in Laos driven by a selective sweep for Plasmodium falciparum kelch13 R539T mutants: a genetic epidemiology analysis
Source: Lancet Infect Dis. 2023 May;23(5):568–77. doi: 10.1016/S1473-3099(22)00697-1 (PMC10914674; doi:10.1016/S1473-3099(22)00697-1)
Supplement: Supplementary appendix 2 [file mmc2.pdf]

THE LANCET
Infectious Diseases

Supplementary appendix 2

This appendix formed part of the original submission and has been peer reviewed. We post it as supplied by the authors.

Supplement to: Wasakul V, Disratthakit A, Mayxay M, et al. Malaria outbreak in Laos driven by a selective sweep for Plasmodium falciparum kelch13 R539T mutants: a genetic epidemiology analysis. *Lancet Infect Dis* 2022; published online Nov 30. https://doi.org/10.1016/S1473-3099(22)00697-1.

Genetic analysis of a malaria outbreak in Laos driven by a selective sweep for *Plasmodium falciparum kelch13* R539T mutants

Supplementary Appendix

Contents

Appendix Tables 3

Appendix Table 1. Number of samples collected in five provinces in Southern Laos. 3

Appendix Table 2. Temporal distribution and genotype summary of 30 clusters identified in Attapeu. 3

Appendix Table 3. Multivariate logistic regression of kelch13 genotypes associated with Attapeu outbreak. 5

Appendix Table 4. Metadata for selected samples used to reconstruct the ancestry of the Attapeu outbreak strains. 5

Appendix Figures 6

Appendix Figure 1. Number of *Plasmodium falciparum* samples in Laos collected by GenRe-Mekong (Apr 2017 - Mar 2021). 6

Appendix Figure 2. Barcode heterozygosity before and during the outbreak across four provinces in Laos. 7

Appendix Figure 3. Prevalence of *kelch13* clusters in Attapeu 8

Appendix Figure 4. Prevalence of the outbreak populations in the Greater Mekong subregion. 8

Appendix Figure 5. Population structure of Attapeu populations shown on PCoA 10

Appendix Figure 6. Contributions to the LAA1 genome by three different genetic sources. 11

Appendix Figure 7. Contributions to the LAA7 genome by two pre-outbreak strains. 11

Appendix Methods 12

Sample and data collection 12

Genotyping and whole-genome sequencing 12

Quality filtering of sample data 13

Pairwise genetic distances based on genetic barcodes 13

Population structure analysis 13

Characterization of population-level genetic diversity 13

Statistical analysis 14

Ancestry analysis 14

Acknowledgements of study sites and local collaborators in the Lao PDR 15

Attapeu Province 15
Champasak Province..15
Salavan Province 16
Sekong Province...17
Savannakhet Province...17

References..19

Appendix Tables

Source	Study	Year	Number of samples before filtering: Attapeu	Champasak	Salavan	Savannakhet	Sekong	Total	Number of samples after filtering: Attapeu	Champasak	Salavan	Savannakhet	Sekong	Total
TRAC [1]	1052-PF-TRAC-WHITE	2011	59					59	59					59
		2012	27					27	26					26
GenRe-Mekong	1208-PF-LA-CMPE-GENRE	2017	97	94	102	272	13	578	86	85	100	259	12	542
		2018	203	139	98	374	9	823	192	136	92	341	8	769
		2019	48	28	27	209	11	323	39	21	15	121	7	203
		2020	210	2	12	64	2	290	157	2	9	53	1	222
		2021	64					64	51					51

Appendix Table 1. Number of samples collected in five provinces in Southern Laos.

The table shows in the first two columns the project and study that contributed the samples; then for each year of collection, it shows the number of samples collected in each of the five provinces, and the total. Numbers are provided for the samples contributed (blue headers), and samples used in analyses after quality filtering (i.e. after removing samples with >25% genotype missingness in the genetic barcodes, yellow headings)

(Next page)

Appendix Table 2. Temporal distribution and genotype summary of 30 clusters identified in Attapeu.

For each cluster, we show: the cluster label; the number of samples per year of collection; the total number of samples in the cluster; and the genotypes/haplotypes at key drug resistance-related loci: *kelch13* (resistance to artemisinin), *plasmepsin 2/3* (piperaquine), *pfcrt* (chloroquine), *pfdhfr* (pyrimethamine); *pfdhps* (sulfadoxine); and *pfmdr1* (amodiaquine). Full details about these haplotypes are given in the SpotMalaria Technical Notes at https://www.malariagen.net/resource/29.

Cluster	2011	2012	2017	2018	2019	2020	2021	Total	Kelch13	Pm2/3	PfCRT	PfDHFR	PfDHPS	PfMDR1
LAA1			3	9	5	108	43	168	R539T:162 ; C580Y:1	WT	CVIET	IRNI	AGEAA	NFD
LAA2			1	1	1	18	4	25	C580Y:22	WT	CVIET	IRNL	SGNGA	NFD
LAA3				25		1		26	WT: 26	WT	CVIDT	IRNI	SAKAA	NYD
LAA4				24	3			27	WT: 27	WT	CVIDT	IRNI	SGEAA	YYD
LAA5			4	18	2	2		26	C580Y:25	Amplified	CVIET	IRNL	SGNGA	NFD
LAA6				8	7	4		19	WT: 19	WT	CVIDT	IRNI	SGKAA	NYD
LAA7					7	8	1	16	R539T:16	WT	CVIET	IRNL	SGNGA	NFD
LAA8			14	3				17	WT:6 ; C580Y:1	WT	CVIDT	IRNI	SGEAA	YYD
LAA9				8	1			9	C580Y:9	Amplified	CVIET	IRNI	SGNGA	NFD
LAA10				10				10	C580Y:10	WT	CVIDT	IRNI	AGEAT	NFD
LAA11			4		3			7	C580Y:5	Amplified	CVIET	IRNL	SGNGA	NFD
LAA12			7					7	C580Y:1	WT	CVIDT	IRNI	AGEAT	NYD
LAA13			7					7	C580Y:5	Amplified	CVIET	IRNL	AGEAA	NFD
LAA14				2	3			5	C580Y:5	Amplified	CVIET	IRNL	SGNGA	NFD
LAA15	2	3						5	WT:5	WT	CVIET	NRNI	AGEAA	NYD
LAA16						4		4	C580Y:4	WT	CVIET	IRNI	SGKAA	NFD
LAA17				4				4	WT:4	WT	CVIDT	IRNI	SAKAA	NYD
LAA18				3				3	C580Y:3	Amplified	CVIET	IRNL	SGNGA	NFD
LAA19				1		2		3	C580Y:3	WT	CVIET	IRNI	SGKAA	NFD
LAA20			4					4	WT:3	WT	CVIDT	IRNI	FAKAS	NYD
LAA21						1	2	3	WT:3	WT	CVIET	IRNI	FAKAS	NFD
LAA22				1		2		3	WT:3	WT	CVIET	IRNI	FAKAS	NYD
LAA23				3				3	WT:3	WT	CVIDT	IRNI	SGKAA	NYD
LAA24				3				3	C580Y:3	Amplified	CVIDT	IRNL	AGEAT	NFD
LAA25				3				3	WT:3	WT	CVIET	IRNI	*G*A*	NYD
LAA26				3				3	C580Y:3	Amplified	CVIET	IRNL	SGNGA	NFD
LAA27				3				3	C580Y:3	Amplified	CVIET	IRNI	AGEAA	NYD
LAA28			2	1				3	WT:2	WT	CVIDT	IRNI	AGEAT	NYD
LAA29			3					3	WT:3	WT	CVIDT	IRNI	AGKAT	NYD
LAA30			3					3		WT	CVIET	IRNI	SAKAA	NYD
Samples in cluster	2	3	52	133	32	150	50	422						
Unique samples	57	23	34	59	7	7	1	188						
Total	*59*	*26*	*86*	*192*	*39*	*157*	*51*	***610***						

Covariate	Odds ratio	z	p Value	95% CI
R539T	52·8	7·055	<0·0001***	19·5-186·0
Het	6·67	1·802	0·072	0·72-54·5
C580Y	1·8	1·037	0·300	0·65-6·41
WT	0·33	-1·726	0·084	0·094-1·29

Appendix Table 3. Multivariate logistic regression of kelch13 genotypes associated with Attapeu outbreak.

For each *kelch13* allele observed in the Attapeu population, we report the odds ratio that it is associated with the outbreak. Other statistics (z-value, p-value and 95% confidence interval) are also reported.

Label	Kelch13	Amplifications	Province, Country	Year	Description	PfCP Release	MalariaGEN Id	ENA Id
**LAA1**	R539T	m+ p-	Attapeu, Laos	2018	Attapeu outbreak strain LAA1	6.3	RCN13530	ERS2866227
**LAA2**	C580Y	m- p-	Attapeu, Laos	2018	Attapeu outbreak strain LAA2	6.3	RCN13540	ERS2866240
**LAA7**	R539T	m- p-	Attapeu, Laos	2019	Attapeu outbreak strain LAA7	6.4	RCN25946	ERR5481228
**KH3**	R539T	m+ p-	Battambang, Cambodia	2008	Early ART-R, KH3 group	6.0	PH0145-CW	ERS024144
**KH2A**	C580Y	m- p-	Battambang, Cambodia	2009	Early ART-R, KH2 group	6.0	PH0147-CW	ERS024146
**KH2B**	C580Y	m- p+	Pursat, Cambodia	2010	Early ART-R, KH2 group (KEL1/PLA1 precursor)	6.0	PH0169-C	ERS014171
**KH3-LA**	R539T	m+ p-	Champasak, Laos	2017	Likely major contributor to LAA1	6.2	RCN08982	ERS2474155
**LAA1-pre**	R539T	m+ p-	Attapeu, Laos	2018	Likely LAA1 precursor, with *mdr1* amplification	6.3	RCN13530	ERS2866227
**LAA1-preWT**	WT	m- p-	Champasak, Laos	2018	Likely contributor to LAA1 (Laos WT parasite)	6.3	RCN13467	ERS2866164
**LAA2-pre**	C580Y	m? p-	Champasak, Laos	2018	Identical to LAA2, found in Laos before outbreak	6.4	RCN15245	ERR3831106
**LAA7-preA**	R539T	m- p-	Attapeu, Laos	2018	Likely contributor to LAA7 (R539T)	6.3	RCN11913	ERS2463794
**LAA7-preB**	C580Y	m- p+	Attapeu, Laos	2018	Likely contributor to LAA7 (C580Y)	6.3	RCN13527	ERS2866224

Appendix Table 4. Metadata for selected samples used to reconstruct the ancestry of the Attapeu outbreak strains.

Whole-genome sequencing data for these samples was used to identify IBD proportions and patterns. For each sample, we show: the label used to refer to the sample in the main text and in Figure 5; the *kelch13* allele; the amplification status for the *pfmdr1* (m) and *plasmepsin 2/3* (p) loci (a + sign indicates amplification, a - indicates single copy, a ? denotes an undetermined result); the province and country of collection; the year of collection; a description of the sample's significance in the ancestry analysis; the *P. falciparum* Community Project release that included the sample; its MalariaGEN identifier; and the sample's identifier at the European Nucleotide Archive (ENA) where the sequencing data is deposited.

Appendix Figures

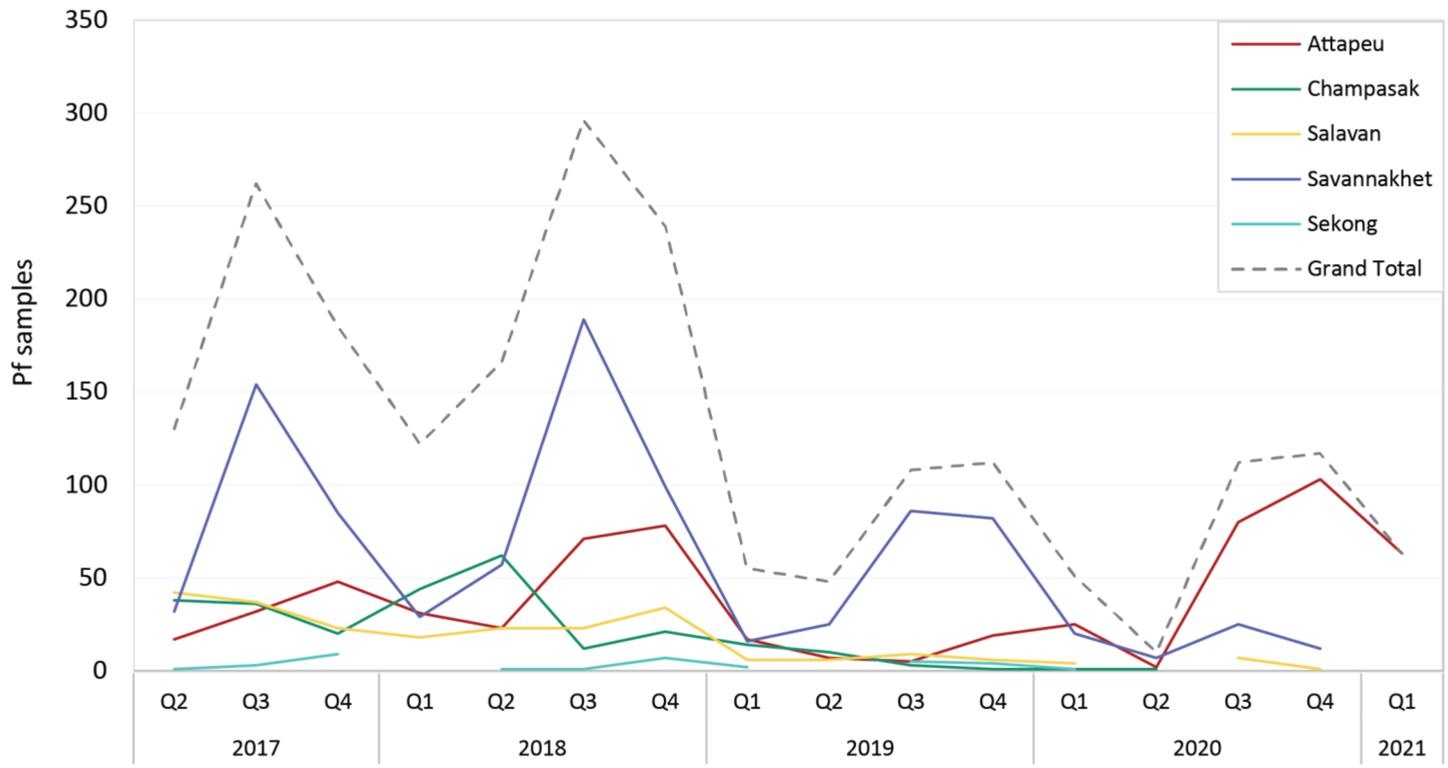

Appendix Figure 1. Number of *Plasmodium falciparum* samples in Laos collected by GenRe-Mekong (Apr 2017 - Mar 2021).

The graph shows the number of samples collected in each quarter in each of the five endemic provinces of Laos. The dashed line shows the total number of samples collected. Q1: January – March; Q2: April – June; Q3: July – September; Q4: October –December.

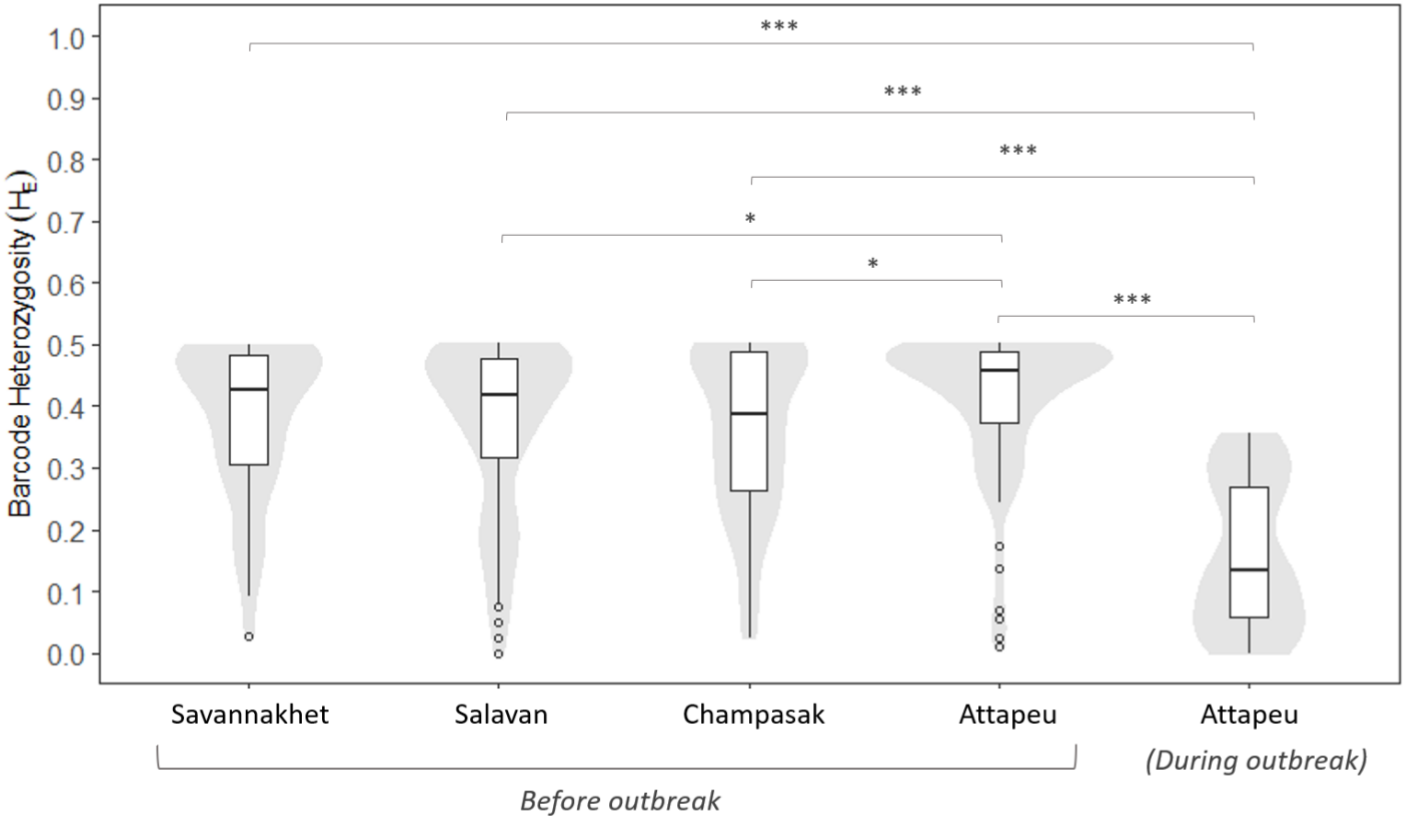

Appendix Figure 2. Barcode heterozygosity before and during the outbreak across four provinces in Laos.

Density plots of the value distributions is shown in grey behind the boxplots. Pairwise comparisons between groups by Wilcoxon rank-sum tests are indicated by horizontal lines and asterisks. Significance codes: *** $p < 0·001$, **$p < 0·01$, *$p < 0·05$. A highly significant reduction in barcode heterozygosity is observed in Attapeu province during the outbreak.

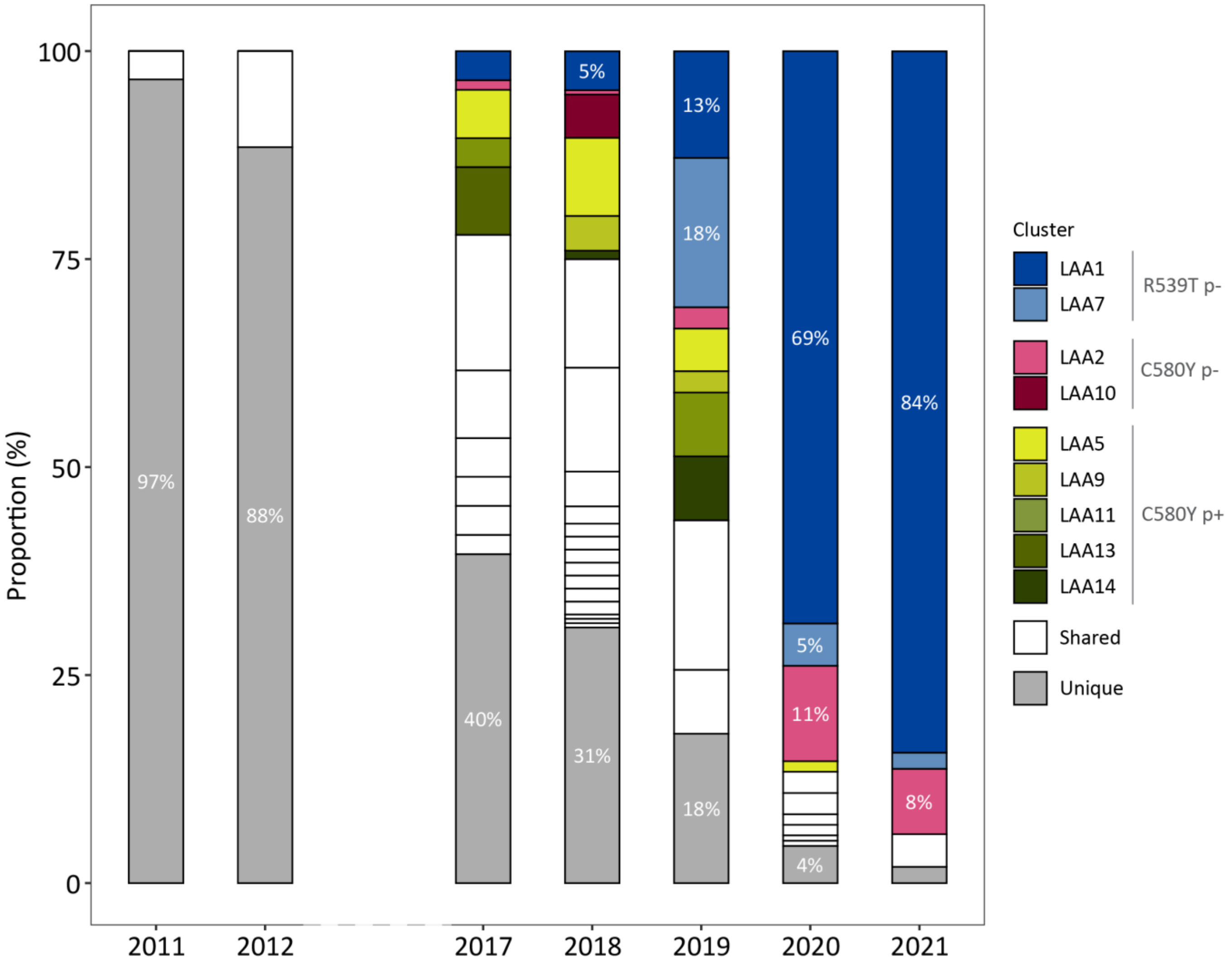

Appendix Figure 3. Prevalence of *kelch13* clusters in Attapeu

Clusters contain samples that share at least 95% barcode identity (see Methods). Colours are given to clusters of at least 5 members in which at least 5 members carry a *kelch13* mutation (R539T or C580Y). Percentage is shown only for LAA1, LAA2 and LAA7 groups when ≥ 4%. As shown in the legend, clusters are grouped according to their *kelch13* genotype and by their *plasmepsin2/3* amplification status (p- and p+ denoting single- and multiple-copy samples), and coloured accordingly. Other clusters (containing *kelch13* wild-type parasites) are not coloured, and labelled as Shared. Parasites that did not form part of any cluster are grouped together in a gray segment, and labelled as Unique.

(Next Page)

Appendix Figure 4. Prevalence of the outbreak populations in the Greater Mekong subregion.

Prevalence and cluster statistic for clusters (A) LAA1, (B) LAA2, and (C) LAA7. Circular markers show the prevalence among all samples from the same province. Provinces are connected by lines whose thickness provides a qualitative visualization of the level of sharing. For LAA1 and LAA7, which are limited to Attapeu province, smaller maps on the right of the panel show the prevalence by district. A large proportion of the population in Attapeu (36%) belongs to the LAA1 cluster, with Phouvong district as the hotspot of transmission. In contrast, LAA2 parasites have been circulating across several GMS countries, albeit at very low frequency.

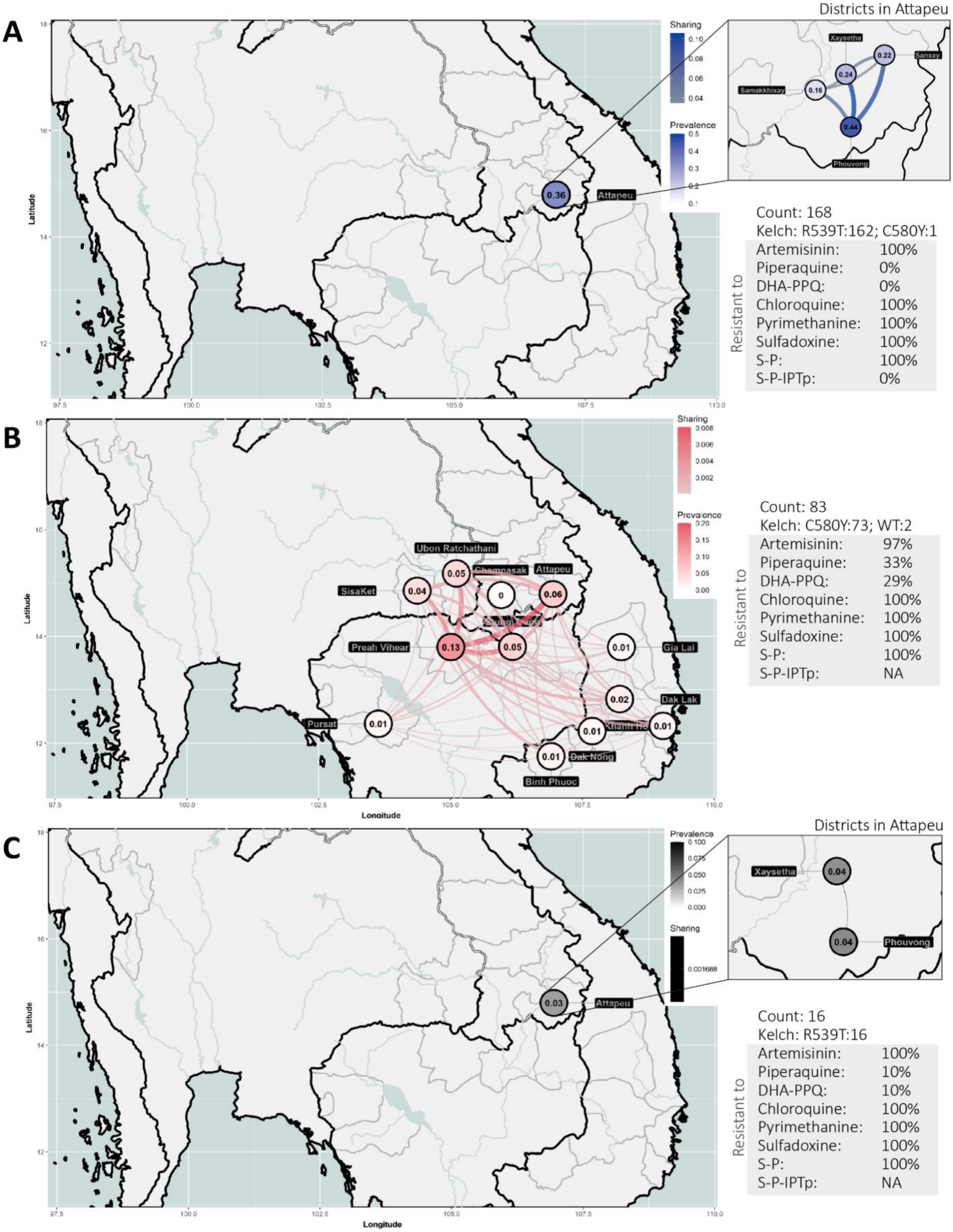
A
Districts in Attapeu
Sharing
Prevalence
Latitude
Xaysetha
Sanxay
Samakkhixay
Phouvong
Attapeu
0.36
0.22
0.24
0.16
0.44
Count: 168
Kelch: R539T:162; C580Y:1
Resistant to
Artemisinin: 100%
Piperaquine: 0%
DHA-PPQ: 0%
Chloroquine: 100%
Pyrimethanine: 100%
Sulfadoxine: 100%
S-P: 100%
S-P-IPTp: 0%
B
Sharing
Prevalence
Latitude
Longitude
Ubon Ratchathani
Champasak
Attapeu
SisaKet
Preah Vihear
Gia Lai
Dak Lak
Pursat
Dak Nong
Binh Phuoc
Count: 83
Kelch: C580Y:73; WT:2
Resistant to
Artemisinin: 97%
Piperaquine: 33%
DHA-PPQ: 29%
Chloroquine: 100%
Pyrimethanine: 100%
Sulfadoxine: 100%
S-P: 100%
S-P-IPTp: NA
C
Districts in Attapeu
Prevalence
Sharing
Latitude
Longitude
Xaysetha
Phouvong
Attapeu
0.03
0.04
0.04
Count: 16
Kelch: R539T:16
Resistant to
Artemisinin: 100%
Piperaquine: 10%
DHA-PPQ: 10%
Chloroquine: 100%
Pyrimethanine: 100%
Sulfadoxine: 100%
S-P: 100%
S-P-IPTp: NA

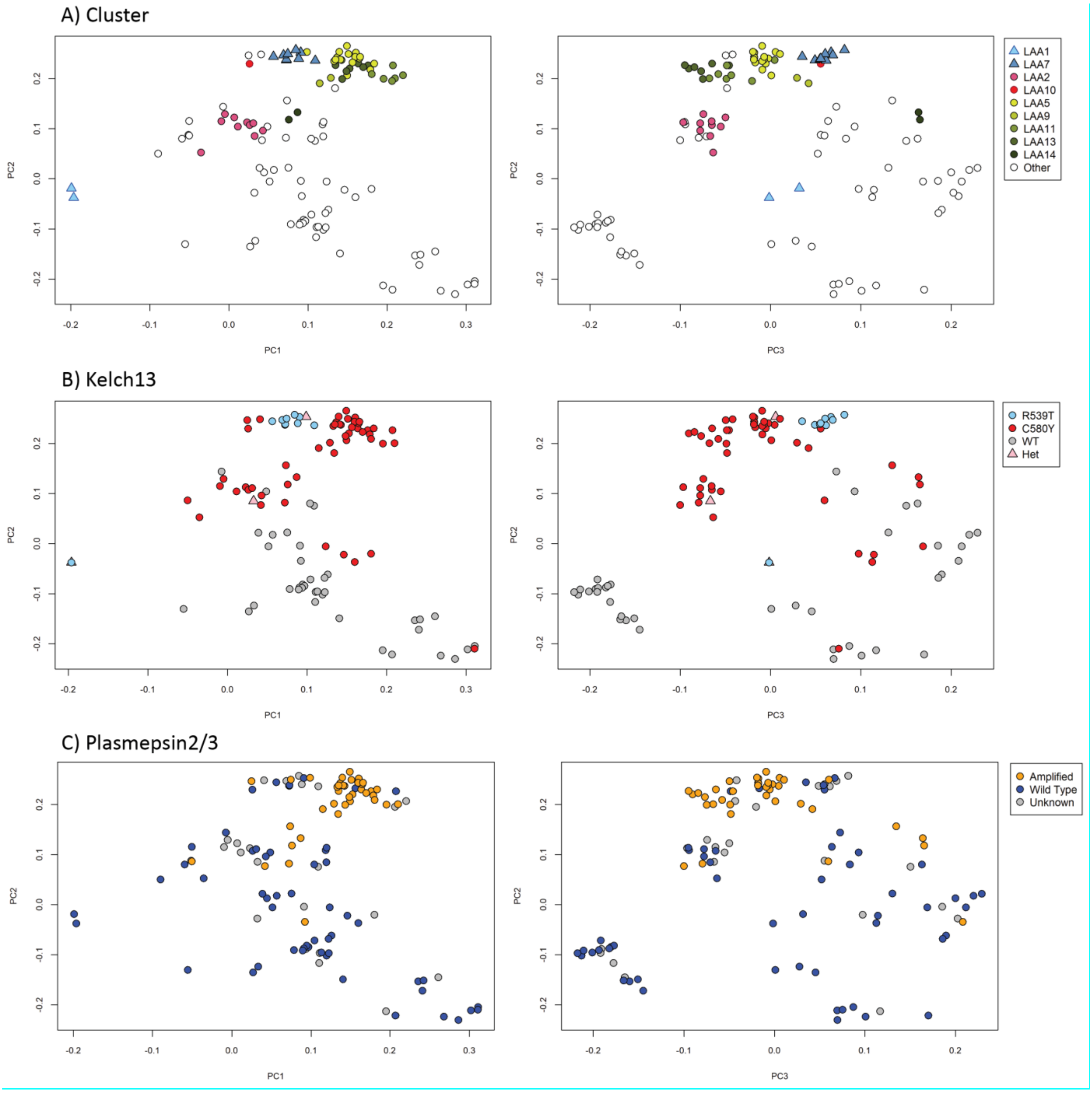

Appendix Figure 5. Population structure of Attapeu populations shown on PCoA

In this PCoA analysis, samples are colored according to (A) their assigned cluster, (B) *kelch13* mutation, and (C) *plasmepsin 2/3* amplifications. Only samples belonging to clusters were included in this analysis. The first three principal coordinates explained 32·2%, 20·6% and 9·5% of the variance, respectively. LAA1 shows clear separation from other clusters and a high degree of identity. Although LAA2 carries the C580Y *kelch13* mutation, it groups separately from earlier KEL1/PLA1 parasites, while LAA7 is more closely related in spite of its R539T mutation.

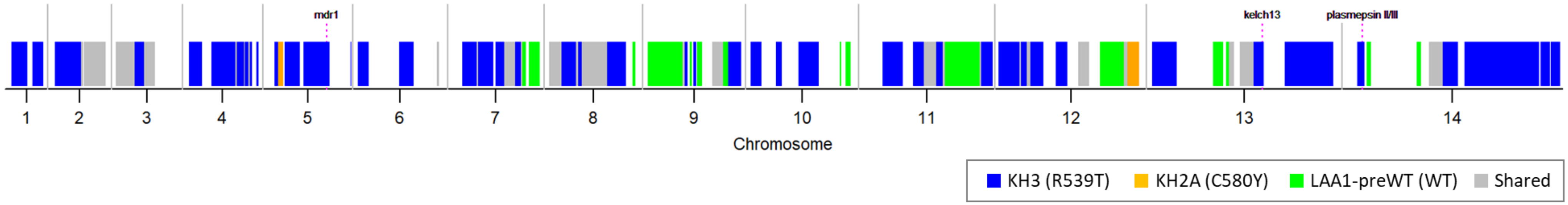

Appendix Figure 6. Contributions to the LAA1 genome by three different genetic sources.

The plot shows the 14 nuclear chromosomes of P. falciparum along the horizontal axis, their size proportional to the number of bases. The chromosomes are separated by gray vertical lines, and dotted lines show the location of the *kelch13* gene and of the *plasmepsin 2/3* and *mdr1* amplifications. Blocks of the LAA1 genome are coloured according to whether they are in IBD with KH3 (blue), KH2A (orange), LAA1-preWT (green) or more than one of these (gray). Regions without a colour block are not in IBD with any of the three strains tested.

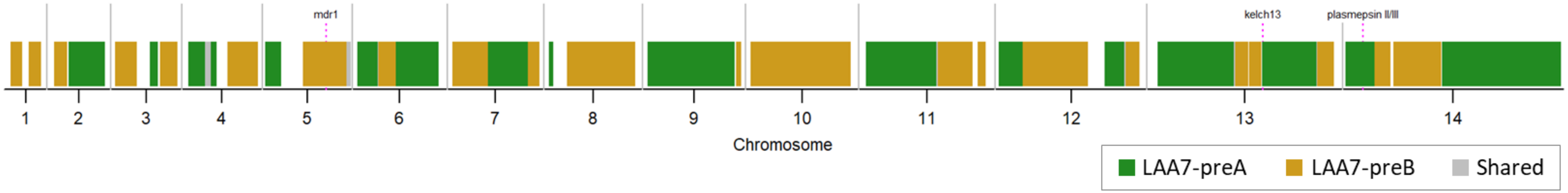

Appendix Figure 7. Contributions to the LAA7 genome by two pre-outbreak strains.

The plot shows the 14 nuclear chromosomes of P. falciparum along the horizontal axis, their size proportional to the number of bases. The chromosomes are separated by gray vertical lines, and dotted lines show the location of the *kelch13* gene and of the *plasmepsin 2/3* and *mdr1* amplifications. Blocks of the LAA7 genome are coloured according to whether they are in IBD with LAA7-preA (R539T, green), LAA7-preB (C580Y, brown) or both (gray). Regions without a colour block are not in IBD with either stra

Appendix Methods

The following sections complement the Methods section in the main paper text.

Sample and data collection

This study was conducted as part of the GenRe-Mekong project for the genetic surveillance of malaria in southern Laos, using a variant of the GenRe-Mekong protocol which is approved by the relevant ethics committees in each country where sample collections are carried out; for further details, refer to the GenRe-Mekong manuscript. [2] For Laos, ethical approvals were obtained from the National Ethics Committee for Health Research (NECHR) of the Health Ministry of the Lao People's Democratic Republic and the Oxford Tropical Research Ethics Committee (OxTREC).

Samples were collected from patients of all ages who has been confirmed positive for *P. falciparum* by rapid diagnostic test or blood smear microscopy at partner public health sites (see Appendix Acknowledgements pp.15-18 for a full list of sites and credits to local collaborators). After obtaining consent, three 20 µl dried blood spots (DBS) on filter paper were obtained from each patient by finger prick, dried and stored in a plastic bag with silica gel. The DBS samples, sample manifests and site records were labelled with scannable unique identification tags. The sample collection date and sample-related information were recorded in the sample manifests and site records. Details of sample collection procedure by GenRe-Mekong has been fully described elsewhere. [2]

All whole-genome sequence (WGS) samples were processed according to the standard MalariaGEN *P. falciparum* Community Project (PfCP), [3] and were genotyped using the PfCP 6.0 variant list.

Genotyping and whole-genome sequencing

Genomic DNA was extracted from dried blood spot using high-throughput robotic equipment (Qiagen QIAsymphony) according to manufacturer's instructions. Parasite DNA was amplified using selective whole genome amplification (sWGA) [4] before targeted genotyping and whole genome sequencing (WGS). Targeted genotyping was performed by the SpotMalaria platform as previously described. [2] For each sample, we produced a Genetic Report Card (GRC) detailing calls for a standardized set of variants and haplotypes relevant to drug resistance, as well as a genetic barcode to enable epidemiological analyses. Full details of GRC contents, and how they are derived through targeted genotyping, are given elsewhere. [2] For samples for which targeted genotyping was not available, GRC variants were called from WGS data using bespoke scripts.

WGS data were obtained using Illumina short-read sequencing technology. Due to lengthier processing times and to operational restrictions during the COVID-19 pandemic, WGS data were not available for samples collected during the later stages of the outbreak. Genotypes were called from WGS reads at 1,042,396 nuclear biallelic single-nucleotide polymorphisms (SNPs) classified as PASS in the PfCP v6.0 variants list (https://www.malariagen.net/data/catalogue-genetic-variation-p-falciparum-v6.0), using the standardised MalariaGEN *Plasmodium falciparum* Community Project genotyping pipeline V6.0. [3]

For a number of samples, we performed copy number estimation for the *plasmepsin2/3* and/or *mdr1* genes by quantitative real-time PCR. The *plasmepsin2/3* amplification was called either by detection of a duplication breakpoint[3] or by qPCR estimation[5]. The *mdr1* amplification was detected either by qPCR estimation[6], or from WGS data using a coverage-based method. [3]

Quality filtering of sample data

As part of the GRC, each sample is assigned a genetic barcode comprising 101 SNP alleles, chosen based on their variability and their power to recapitulate genetic distance. [2] Before proceeding with epidemiological analyses, we performed a quality filtering step to reduce errors due to missingness in the data (i.e. positions where a genotype could not be called). First, we removed samples with more than 50% missing barcode genotypes. Then, we filtered out barcode SNPs with missing calls in more than 25% of the remaining samples. Lastly, we removed samples with more than 25% missing calls in the remaining barcode SNPs.

Pairwise genetic distances based on genetic barcodes

To identify clusters of highly related parasites and provide input for population structure analysis, we computed pairwise genetic distances between relevant pair of samples. For each sample, a within-sample non-reference genotype frequency (g_s) was assigned at each barcode position. If the sample carried the reference allele, 0 is assigned; if the sample carried the alternative allele, 1 is assigned; if both alleles were present, 0·5 is assigned. Distance between two samples at that position was then calculated using the equation: $d = g_1(1 - g_2) + g_2(1 - g_1)$, where g_1 and g_2 is the genotype frequency of sample 1 and sample 2 respectively. The pairwise distance was calculated as the mean of d across all loci where neither of the two samples had a missing call.

Population structure analysis

Samples collected in Attapeu between January 2011 and April 2021 were assigned into clusters of three or more similar parasites. Clustering was based on their pairwise genetic barcode similarity s, obtained from the pairwise genetic distance d where s=(1-d). Using the igraph R package[7] (https://igraph.org/r/). We constructed a graph connecting sample pairs with s greater than a minimum threshold s_{min}, and applied the Louvain multilevel community detection algorithm[8] to partition the graph into clusters. In the present analysis, we used s_{min}=0·95 to obtain clusters of parasites with essentially identical barcodes, allowing for a low proportion of genotyping errors. To assess whether the outbreak clusters were present outside of southern Laos, we also performed clustering after including publicly available GRC data from 9,609 samples collected from eight countries by the GenRe-Mekong Project[2].

To visualize relationships between samples, heatmaps of pairwise distance matrices were produced using the `Heatmap` function in the ComplexHeatmap R package (V2.8.0). [9] The arrangement of samples on the heatmap was determined by hierarchical clustering using the shortest distance between two samples (`clustering_distance='euclidean'`) to maximize the distance between two clusters (`clustering_method='complete'`). Annotations were passed onto the heatmap to provide sample metadata including: collected year, *kelch13* mutation, *plasmepsin2/3* amplification status, and cluster label or country of collection. The same pairwise genetic distance matrix was used to perform principal coordinate analysis (PCoA) using the `cmdscale` function of the ape R package. [10] Samples that did not cluster with any other sample were excluded in the PCoA.

Characterization of population-level genetic diversity

Population-level genetic diversity was assessed by the mean expected heterozygosity $\overline{H}_E$ across all barcode SNPs. At each barcode SNP, the expected heterozygosity H_E (the proportion of heterozygous genotypes expected under Hardy-Weinberg equilibrium[11]) was estimated using the equation: $H_E = \frac{n}{n-1}\left[1 - \sum_i {p_i}^2\right]$, where n = the number of samples in the population that carried a valid genotype at this SNP, and p_i = the allele frequency of the ith allele observed. Missing alleles

were removed before computing allele frequencies (p_i) and heterozygous alleles contributed by 0.5 to each of the nucleotide allele in the locus.

Statistical analysis

All statistical analyses were performed using R (V4.2.0). For pairwise comparison between groups we used Wilcoxon rank-sum tests with a method to adjust *P* value for controlling false discovery rate, using `pairwise.wilcox.test(x, p.adjust.method = "fdr")` function. For comparison between multiple populations or subgroups, we used `kruskal.test` function to perform Kruskal-Wallis tests. *P* values <0·05 were considered statistically significant.

To determine which *kelch13* genotypes were associated with the outbreak in Attapeu, covariates were entered into a logistic regression model. Linear regression was performed using `glm` function in R, with outbreak outcomes as the dependent variable and *kelch13* genotypes as the predictors. Exponentiating the linear regression coefficient provided the odds ratio, which gives a measure of the impact of changing a *kelch13* genotype on the odds of an outbreak occurring.

Ancestry analysis

Using genotypes obtained from WGS data for all PfCP samples from Laos, Cambodia, Vietnam and northeast Thailand, we identified 53,150 SNPs for which there was at least one sample with a homozygous call for the reference allele, and at least one with a homozygous call for an alternative allele. We used these genotypes to estimate alternative allele frequencies; missing genotypes were excluded in this estimation. Identity by descent (IBD) analysis was performed on the resulting genotypes and allele frequencies, using hmmIBD[12] with default parameters. Visualizations of recombination patterns were produced from hmmIBD results using bespoke R scripts.

Acknowledgements of study sites and local collaborators in the Lao PDR

We thank the following individuals at the study sites in the Lao PDR for their input to the present outbreak study as well as for their contributions to the GenRe-Mekong Project.

Attapeu Province

Collaborators

Mr. Sengdala Phetsomphou	Mr.Philavong Dethphachan
Mrs. Khamlar Khounxay	Mr. Bounkan Vongsamlan
Mr. Phou Ngeun Xaiyavong	Mr. Kethsana Xaiyasaeng
Mr. Boudmanda Vongphanu	Mr. Simsa Nguan Saenthuliboud
Ms. Ari Phommachan	Mrs. Nuthong Sibounhueng
Mrs. Nali Xaiyaserm	Ms. Oudomhak Singsombus
Mr. Phouvieng Xinalath	Mr. Kawin Xaybounmee

Study sites

LA500 Attapeu PH, Attapeu, Attapeu
LA502 Attapeu Provincial Military Hospital, Attapeu, Attapeu
LA510 Xaysetha DH, Xaysetha, Attapeu
LA511 Keng Yai HC, Xaysetha, Attapeu
LA512 Phon Gnam HC, Xaysetha, Attapeu
LA513 KhengMak-Keun HC, Xaysetha, Attapeu
LA514 PhaoSamPhanMeeXay HC, Xaysetha, Attapeu
LA515 SaPhouan HC, Xaysetha, Attapeu
LA520 Sanamxay DH, Sanamxay, Attapeu
LA521 Somsouk HC, Sanamxay, Attapeu
LA522 Sompoy HC, Sanamxay, Attapeu
LA530 Xanxay DH, Xanxay, Attapeu
LA540 Phouvong DH, Phouvong, Attapeu
LA541 Phouhome HC, Phouvong, Attapeu

Champasak Province

Collaborators

Mr. Saitadam Phetxilar	Mrs. Kularb Keokounmuong
Mrs. Aran Souniyavong	Mr. Sam Keomanivong
Mrs. Phetsamon Sisavath	Mrs. Amphon Keomanee
Mrs. Kanhmany Vongphoumee	Mr. Phoukhanh Chanthavisin
Mrs. Phaitoun Inthathilath	Mrs. Kaikham Inthathirath
Mr. Toulee Saivaya	Mr. Chanthala Sisomvang
Mrs. Vilayvanh Vongphoumee.	Mr.Thongbai Phommixay
Mr. Khamphong Luangphasee	Mrs. Soutthanong Sisamoud
Mr. Amphon Vongsounthone	Mrs. Vanthong Somchidkhamluxay
Mrs. Soviet Pasidson	Mrs. Navalit Sivilay
Mr. Shai Namxokchid	

Study sites

LA300 Pakse PH, Pakse, Champasak
LA303 Champasak Provincial 106 Technical of Military Hospital, Pakse, Champasak
LA310 Paksong DH, Paksong, Champasak
LA311 Pakpong HC, Paksong, Champasak
LA312 Huaykong HC, Paksong, Champasak
LA320 Pathoumphone DH, Pathoumphone, Champasak
LA321 Phapho HC, Pathoumphone, Champasak
LA322 Sanod HC, Pathoumphone, Champasak
LA323 Palai HC, Pathoumphone, Champasak
LA330 Mounlapamok DH, Mounlapamok, Champasak
LA331 Nong nga HC, Mounlapamok, Champasak
LA332 Nadee HC, Mounlapamok, Champasak
LA340 Khong DH, Khong, Champasak
LA341 Nafang HC, Khong, Champasak
LA342 Phonsa arth HC, Khong, Champasak
LA343 Ban Soth HC, Khong, Champasak
LA350 SaNaSomBoun DH, SaNaSomBoun, Champasak
LA360 ChamPaSak DH, ChamPaSak, Champasak
LA361 NongTae HC, ChamPaSak, Champasak
LA370 SouKhoumMa DH, SouKhoumMa, Champasak

Salavan Province

Collaborators

Mr. Keomany	Mr. Lamphanh Salapheung
Mrs. Kaikeo Duongxilee	Mr. Bounlom Phonsaly
Mrs. Keochai Xaiyasid	Mr. Khammanh Phommixay
Ms. Khamkak Xaiyason	Mr. Somchit Keochomlath
Mrs. Vilayvong Sengkeonee	Mrs. SomThid Molixath
Mrs. Eatsaphone Duongphasouk	Mrs. Kanha Souliya
Mr. Mad Khamsouk	Mrs. Singha
Ms. Ximuon Xixana	Mr. Kuta Keotokong
Mrs. Chanthaly Soukhamthud	Mr. Boualapha Khamcounmuong
Mr. Phetsamai Getsombus	Mr. Savanthong Keoduongsee

Study sites

LA200 Salavan PH, Salavan, Salavan
LA202 Salavan Provincial Military Hospital, Salavan, Salavan
LA210 Ta Oy DH, Ta Oy, Salavan
LA211 Phoutang HC, Ta Oy, Salavan
LA212 Pajudone HC, Ta Oy, Salavan
LA213 SoyTamh HC, Ta Oy, Salavan
LA214 KokBok HC, Ta Oy, Salavan
LA220 Toomlarn DH, Toomlarn, Salavan
LA221 Tabeng HC, Toomlarn, Salavan
LA230 Vapy DH, Vapy, Salavan
LA231 Khonsai HC, Vapy, Salavan
LA232 Saphab HC, Vapy, Salavan
LA240 Samuoi DH, Samuoi, Salavan
LA241 Asok HC, Samuoi, Salavan
LA242 Axing HC, Samuoi, Salavan
LA243 KiNae HC, Samuoi, Salavan
LA244 AhWao HC, Samuoi, Salavan
LA245 Khongsedone DH, Khongsedone, Salavan
LA246 Lakhonepheng DH, Lakhonepheng, Salavan

Sekong Province

Collaborators

Mrs. Sonsamai Xaiyavong	Mr. Siamphai Canlamphan
Mrs. Thidthisouk Kongsavanh	Mr. Huck Kampouxa
Mr. Phoudsy Xaysueksa	Mrs. Jhonlakham Soulivanh
Mrs. Sengkham Phonvilay	Mr. SantiPhone Xaiyason
Mrs. Daovaly Dalavong	Mr. Kamxi Vongvaengouk
Mr. Phou Ngeun Doungmala	Mr. SanDey Leuymalaysee
Mrs. Orlaphan Souvannasee	Mrs. Sidda Amphaiphon
Mrs. Vanthana Inthamixay	Mr. Sida Inlavong

Study sites

LA400 Sekong PH, Sekong, Sekong

LA402 Sekong Provincial Military Hospital, Lamam, Sekong

LA410 Thateng DH, Thateng, Sekong

LA411 Thonnoy HC, Thateng, Sekong

LA420 Kaleum DH, Kaleum, Sekong

LA421 Jii HC, Kaleum, Sekong

LA430 Dakcheung DH, Dakcheung, Sekong

LA431 Dakdun HC, Dakcheung, Sekong

LA442 Ban Phone HC, Lamam, Sekong

LA443 NaKhasangkang HC, Lamam, Sekong

LA444 TaNeup HC, Lamam, Sekong

LA445 Tock Ongkeo HC, Lamam, Sekong

LA446 DoneChanh HC, Lamam, Sekong

LA447 Ban Ngeup HC, Lamam, Sekong

Savannakhet Province

Collaborators

Mrs. Vilayphone Pha Arnan	Mrs. Phansida chanthilanong
Mrs. Phaivanh Sykhammounty	Mrs. Nilaphon OunLeuyvongsak
Mrs. Vanna Saengchan	Mrs. Nui Lianvilay
Mrs. Souksavanh Khambonvilay	Mrs. Malina Xaiyavong
Mrs. Outhan Xiphommalangkoun	Ms. Sompharn Vannavong
Mr. Xisouphan Luangphasee	Ms. Phoukham Malavanh
Mrs. Khammany Saenboudpalath	Mr. Kongkeo Thammalin
Ms. Rodjana Boudsisavanh	Mr. Souksamon Thinlanong
Ms. Phouthon Souliyavong	Mrs. Sengsavanh Harnxana
Ms. Phoukhao Phudthavadee	Mrs. Leevon Xaiyacom
Mr. Chai Khamphiu	Mr. Bounhom Chanthaluxay
Ms. Authai Lukhamharn	Mr. Norchan Keoboaphan
Ms. Kayson Sisaketkhammuon	Mr. Lounnee Xaiyavong
Mrs. Toukta Khankeo	Ms. Vivanxay Silathom
Mr. Sonphet Sidala	Ms. Immalar Lathxavongphon
Ms. Intong Saengsoulichan	Mr. Khamkai Phetluangsee
Mr. Vongphachan Bounmee	Mr. Bualom Bangmano
Ms. Souvanna Ludthaphon	Mrs. Ketsoudar Duongsavanh

Study sites

LA100 Savannakhet PH, Savannakhet, Savannakhet
LA102 Savannakhet Provincial Military Hospital, Kaysone, Savannakhet
LA110 Phine DH, Phine, Savannakhet
LA111 Nathong HC, Phine, Savannakhet
LA112 Maiphousy HC, Phine, Savannakhet
LA113 Tang-Alai HC, Phine, Savannakhet
LA114 TounKamh HC, Phine, Savannakhet
LA115 HouyHoi HC, Phine, Savannakhet
LA120 Sepone DH, Sepone, Savannakhet
LA121 Phohai HC, Sepone, Savannakhet
LA122 Phabang HC, Sepone, Savannakhet
LA123 Dansavan HC, Sepone, Savannakhet
LA124 DongSaVanh HC, Sepone, Savannakhet
LA125 ManhChee HC, Sepone, Savannakhet
LA127 KhaToub HC, Sepone, Savannakhet
LA130 Nong DH, Nong, Savannakhet
LA131 Nakong HC, Nong, Savannakhet
LA132 Dongnasan HC, Nong, Savannakhet
LA133 TangAhLai HC, Nong, Savannakhet
LA134 LaKhai HC, Nong, Savannakhet
LA135 KhaySone HC, Nong, Savannakhet
LA136 PhounMarkMee HC, Nong, Savannakhet
LA137 AhSing HC, Nong, Savannakhet
LA138 DaenViLay HC, Nong, Savannakhet
LA140 Thapanthong DH, Thapanthong, Savannakhet
LA141 Sepong HC, Thapanthong, Savannakhet
LA142 Xekeu HC, Thapanthong, Savannakhet
LA143 Phoumaly HC, Thapangthong, Savannakhet
LA144 Khathongneun HC, Thapangthong, Savannakhet
LA150 Vilabuly DH, Vilabuly, Savannakhet
LA151 Nammahi HC, Vilabuly, Savannakhet
LA160 Thaphalanxay DH, Phalanxay, Savannakhet
LA170 Nachantai HC, Phalanxay, Savannakhet
LA171 Nakapong HC, Phalanxay, Savannakhet
LA172 Songkhone DH, Songkhone, Savannakhet
LA190 Xonboury DH, Xonboury , Savannakhet

References

1. Ashley EA, Dhorda M, Fairhurst RM, et al. Spread of Artemisinin Resistance in *Plasmodium falciparum* Malaria. New England Journal of Medicine 2014; 371(5): 411-23.

2. Jacob CG, Thuy-Nhien N, Mayxay M, et al. Genetic surveillance in the Greater Mekong subregion and South Asia to support malaria control and elimination. Elife 2021; 10.

3. MalariaGEN, Ahouidi A, Ali M, et al. An open dataset of *Plasmodium falciparum* genome variation in 7,000 worldwide samples. Wellcome Open Res 2021; 6: 42.

4. Oyola SO, Ariani CV, Hamilton WL, et al. Whole genome sequencing of *Plasmodium falciparum* from dried blood spots using selective whole genome amplification. Malar J 2016; 15(1): 597.

5. Ansbro MR, Jacob CG, Amato R, et al. Development of copy number assays for detection and surveillance of piperaquine resistance associated plasmepsin 2/3 copy number variation in *Plasmodium falciparum*. Malar J 2020; 19(1): 181.

6. Price RN, Uhlemann AC, Brockman A, et al. Mefloquine resistance in *Plasmodium falciparum* and increased pfmdr1 gene copy number. Lancet 2004; 364(9432): 438-47.

7. Csardi G, Nepusz T. The igraph software package for complex network research. InterJournal 2006; Complex Systems: 1695.

8. Blondel VD, Guillaume J-L, Lambiotte R, Lefebvre E. Fast unfolding of communities in large networks. Journal of Statistical Mechanics: Theory and Experiment 2008; 2008(10): P10008.

9. Zuguang Gu RE, Matthias Schlesner. Complex heatmaps reveal patterns and correlations in multidimensional genomic data. Bioinformatics 2016.

10. Paradis E, Schliep K. ape 5.0: an environment for modern phylogenetics and evolutionary analyses in R. Bioinformatics 2019; 35(3): 526-8.

11. Nei M. Analysis of gene diversity in subdivided populations. Proceedings of the National Academy of Sciences of the United States of America 1973; 70(12): 3321-3.

12. Schaffner SF, Taylor AR, Wong W, Wirth DF, Neafsey DE. hmmIBD: software to infer pairwise identity by descent between haploid genotypes. Malar J 2018; 17(1): 196.
